# Identification and verification of disulfidptosis-related genes in sepsis-induced acute lung injury

**DOI:** 10.3389/fmed.2024.1430252

**Published:** 2024-08-28

**Authors:** Anqi Zhang, Xinyang Wang, Wen Lin, Haoqi Zhu, Jingyi Pan

**Affiliations:** ^1^Department of Anesthesiology, The First Affiliated Hospital of Wenzhou Medical University, Wenzhou, China; ^2^Department of Anesthesiology, Fujian Province Second People's Hospital, The Second Affiliated Hospital of Fujian University of Traditional Chinese Medicine, Fuzhou, China; ^3^Department of Gastroenterology, Wenzhou Central Hospital, Wenzhou, China

**Keywords:** disulfidptosis, sepsis, immune infiltration, network analysis, Mendelian randomization

## Abstract

**Background:**

Sepsis-induced acute lung injury (ALI) is a common and serious complication of sepsis that eventually progresses to life-threatening hypoxemia. Disulfidptosis is a newly discovered type of cell death associated with the pathogenesis of different diseases. This study investigated the potential association between sepsis-induced acute lung injury and disulfidptosis by bioinformatics analysis.

**Methods:**

In order to identify differentially expressed genes (DEGs) linked to sepsis, we screened appropriate data sets from the GEO database and carried out differential analysis. The key genes shared by DEGs and 39 disulfidptosis–related genes were identified: ACSL4 and MYL6 mRNA levels of key genes were detected in different datasets. We then used a series of bioinformatics analysis techniques, such as immune cell infiltration analysis, protein–protein interaction (PPI) network, genetic regulatory network, and receiver operating characteristic (ROC), to investigate the possible relationship between key genes and sepsis. Then, experimental verification was obtained for changes in key genes in sepsis-induced acute lung injury. Finally, to investigate the relationship between genetic variants of MYL6 or ACSL4 and sepsis, Mendelian randomization (MR) analysis was applied.

**Results:**

Two key genes were found in this investigation: myosin light chain 6 (MYL6) and Acyl-CoA synthetase long-chain family member 4 (ACSL4). We verified increased mRNA levels of key genes in training datasets. Immune cell infiltration analysis showed that key genes were associated with multiple immune cell levels. Building the PPI network between MYL6 and ACSL4 allowed us to determine that their related genes had distinct biological functions. The co-expression genes of key genes were involved in different genetic regulatory networks. In addition, both the training and validation datasets confirmed the diagnostic capabilities of key genes by using ROC curves. Additionally, both *in vivo* and *in vitro* experiments confirmed that the mRNA levels of ACSL4 and MYL6 in sepsis-induced acute lung injury were consistent with the results of bioinformatics analysis. Finally, MR analysis revealed a causal relationship between MYL6 and sepsis.

**Conclusion:**

We have discovered and confirmed that the key genes ACSL4 and MYL6, which are linked to disulfidptosis in sepsis-induced acute lung injury, may be useful in the diagnosis and management of septic acute lung injury.

## 1 Introduction

Sepsis is a systemic syndrome, which characteristic of systemic organ damage caused by dysregulation of the response of the host to infection. The systemic inflammatory response can lead to disseminated intravascular coagulation (DIC), multiple organ dysfunction syndrome (MODS), and even death (1).

Sepsis-induced acute lung injury (ALI) is a common and severe complication of sepsis characterized by diffuse alveolar injury and pulmonary vascular hyperpermeability (2), it will evolve into life-threatening hypoxemia eventually. Although the understanding of pathophysiology and treatment of sepsis–induced ALI has advanced significantly over the past few decades, it remains a significant cause of mortality for intensive care unit patients all around the world (3). Therefore, early diagnosis and intervention are particularly important for septic patients, and there is an urgent need to study new biomarkers to identify and validate the disease.

Regulated cell death (RCD) is a physiological process to maintain biological development and internal environment stability (4, 5). Disulfidptosis, a newly defined form of cell death belonging to RCD, was recently discovered by Liu et al (6). Disulfidptosis is the name given to the new type of cell death because it cannot be stopped by removing the necessary genes for apoptosis or iron death nor can it be inhibited by generic cell death inhibitors. Instead, thiol oxidants such as diamide and diethyl maleate can greatly accelerate disulfidptosis. The early investigation discovered that in glucose-starved cells with overexpressed SLC7A11, NADPH was markedly reduced. Then, an increase in the content of disulfide bonds in the actin cytoskeleton causes disulfide bond crosslink of actin cytoskeleton protein, contraction of actin filaments, and the collapse of the cytoskeleton structure. Ultimately, this leads to the actin network disintegration and rapid cell death. Currently, many diseases, such as neurodegenerative diseases, cancer, and sepsis, can be treated by targeted intervention in specific cell death pathways (7). The discovery of disulfidptosis surely offers a new potential therapeutic target for a variety of diseases. According to the studies mentioned above, contraction of actin filaments in disulfidptosis leads to the loss of intercellular adhesion and endothelial and epithelial cell hyperpermeability, both of which are critical factors in the disease progression of sepsis-induced ALI (2). To date, the connection between disulfidptosis and sepsis-induced ALI has not yet been investigated.

The objective of this study was to investigate the target of disulfidptosis in sepsis-induced ALI as well as any possible connections or mechanisms. Following an investigation of the Gene Expression Omnibus (GEO) database to find the differentially expressed genes (DEGs) in sepsis, further analysis was performed to discover disulfidptosis-related DEGs by extracting the DEGs in sepsis and disulfidptosis-related genes (DRGs). Disulfidptosis-related DEGs included Acyl-CoA synthetase long-chain family member 4 (ACSL4) and myosin light chain 6 (MYL6), which were regarded as key genes subsequently. Thereafter, we used kinds of bioinformatics analysis methods, including functional enrichment analysis, immune cell infiltration and correlation analysis, network analysis, and MR analysis to explore the existence of genetic regulatory networks, associated functions, and the diagnostic significance of the key genes in sepsis-related ALI. In addition, some of the results of bioinformatics analysis are further supported by basic experiments.

## 2 Materials and methods

### 2.1 Data collection and description

Four microarray datasets of sepsis were obtained from the Gene Expression Omnibus (GEO) database.^1^ The GSE26378 set includes 82 sepsis patients and 21 healthy controls. The GSE28750 set includes 10 sepsis patients and 20 healthy controls. The GSE65682 set includes 479 sepsis patients and 42 healthy controls. These three datasets were included in the training dataset. The GSE95233 set, which contains 102 sepsis patients and 22 healthy samples, was selected as a validation dataset to verify the reliability of our results. The basic details of the above datasets are shown in Table 1. Thirty nine Disulfidptosis–related genes were obtained from the literature (shown in Supplementary Table S1) (6, 8, 9).

**Table 1:** Detailed information on the training and validation datasets.

Date type	Accession	Organism	Platform	Experiment type	Tissue	Groups	
						Control *n*	Sepsis *n*
Training dataset	GSE26378	*Homo sapiens*	GPL570	Array	Whole blood	21	82
	GSE28750	*Homo sapiens*	GPL570	Array	Whole blood	20	10
Validation dataset	GSE65682	*Homo sapiens*	GPL13667	Array	Whole blood	42	479
	GSE95233	*Homo sapiens*	GPL570	Array	Whole blood	22	102

### 2.2 Identification of DEGs and key genes

To identify the DEGs between control groups and sepsis, we used the online analysis tool named GEO2R,^2^ which is an R-based web application included in the GEO database. The Benjamini and Hochberg methods were used to minimize the false-positive rate by employing adjusted *p*-values. ∣log2 FC | ≥ 1 and *p* < 0.05 were set to be the DEGs screening threshold. The DEGs derived from the datasets were processed by GraphPad software and shown as volcano plots. Venn diagrams were used to screen the downregulated and upregulated genes in different datasets and identify disulfidptosis-related DEGs. Among them, disulfidptosis-related DEGs were considered as key genes between sepsis and disulfidptosis.

### 2.3 Immune cell infiltration and correlation analysis

To predict the relative proportion of immune cell infiltration, we used the CIBERSORT algorithm to calculate the abundance of 22 types of immune cell infiltration. This part of the results was obtained from the CIBERSORTx^3^ online website and visualized using origin software. Boxplot of immune-associated cells proportion between sepsis and control groups was drawn using R software and compared via Wilcoxon rank sum test. Moreover, we evaluated the correlation between disulfidptosis-related DEGs and infiltrating immune cells by performing Spearman’s correlation analysis and visualizing by R software.

### 2.4 GO and KEGG biological functions enrichment analysis

The GeneMANIA database^4^ was utilized to create the protein–protein interaction (PPI) network between ACSL4 and MYL6. Then, Gene Ontology (GO) enrichment analysis and the Kyoto Encyclopedia of Genes and Genomes (KEGG) pathway enrichment analysis of related genes were performed by the Metascape database,^5^ choosing the first four significant biological functions from each enrichment analysis and using Origin2021 to create the Sankey bubble chart.

### 2.5 Co-expressed genes and their genetic regulatory network of key genes associated with sepsis

ACSL4 and MYL6 co-expression genes were identified from the Coexpedia dataset. Evenn^6^ was used to show the overlapping genes by creating a Venn diagram. The protein–protein interaction (PPI) network was analyzed by the IMEx interactome database. The Transcript factor (TF)–microRNA (miRNA) coregulatory interaction information was acquired from the RegNetwork repository. The NetworkAnalyst3.0^7^ platform facilitated the identification of networks that interact with key genes. The interaction relationships of these genes were visualized by Cytoscape software.

### 2.6 Receiver operating characteristic curves

To evaluate the predictive accuracy for sepsis of the identified key genes, we validated the ROC curves by using GraphPad Prism 8.0. Then, the area under the curve (AUC) value, cutoff value, sensitivity, and specificity of the ROC curve were calculated.

### 2.7 Animals and experimental groups

Shanghai SLAC Laboratory provided male C57BL/6 mice, aged between 6 and 8 weeks. In the course of the research, each mouse was kept in a specific pathogen-free environment and was able to eat normally, where temperatures were maintained at 22–24°C, humidity controlled at 50–60%, and ray cycles controlled at 12:12. Two groups, one for the lipopolysaccharide (LPS) group and the other for the Sham group, were randomly assigned to the mice (*n* ≥ 3). Mice in the LPS group were intraperitoneally injected with 10 mg/kg LPS to establish a mouse sepsis model. After 24 h, the mice were euthanized and lung tissue samples were taken. The study was authorized by the First Affiliated Hospital of Wenzhou Medical University’s Animal Studies Ethics Committee (WYYY-AEC-YS-2023-0538).

### 2.8 Cell and experimental groups

Purchased from the ATCC in Manassas, United States, RAW264.7 macrophages were cultivated in DMEM supplemented with 10% FBS. The cells were grown in an incubator with 5% CO2 at 37°C. Two groups of cells—one for the LPS group and the other for the control group—were randomly assigned. The LPS group was treated by 500 ng/mL LPS for 24 h.

### 2.9 Histopathology

Lower lobes of the right lung of mice were paraffin-embedded, fixed for 24 h, and weighed. Hematoxylin–eosin (H&E) was used to stain the paraffin sections. Under a microscope, lung tissue damage was seen. The lung damage score was assessed using a double-blind technique based on the pathological alterations found in the lung histology. When observing pathological sections with microscope, the lung injury score was made according to the thickness of alveolar wall/hyaline membrane, alveolar congestion/bleeding, infiltration level of inflammatory cells: 0 = no injury; 1 = mild injury (25%); 2 = moderate injury (50%); 3 = serious injury (75%); 4 = much more serious injury (almost 100%), and the score of each pathological change is 0–4. Add these four scores to determine the lung injury score (total score: 0–16).

### 2.10 Quantitative real-time polymerase chain reaction

TRIzol (Invitrogen, Carlsbad, California, United States) was used to extract the total RNA of the RAW264.7 cells and lung tissue. Reverse transcription was performed using the TaqMan^™^ Reverse Transcription Kit (Thermo Fisher), and qRT-PCR was performed using the PowerTrack™ SYBR Green Kit (Thermo Fisher). Primer sequences for the detection of normalizing the expression of the target genes, β-actin/GAPDH served as an internal reference gene. The relative gene expression levels were calculated with the 2^(−△△CT)^ method.

### 2.11 Measurement of NADH+/NADPH, G6P, and G6PDH indicators

Tissues and cells were collected, and the NADH+/NADPH (S0179) ratio, G6P content (S0185), and G6PDH activity (S0189) in tissues and cells were measured according to the instructions of the reagent kit.

### 2.12 Mendelian randomization analyses

To determine the cause-and-effect relationship between the illnesses and key gene expression levels, MR was used. Instrumental variables (Ivs) were defined as single-nucleotide polymorphisms (SNPs). The publically accessible genome-wide association study (GWAS) databases provided the gene data. The participants of the European population provided the exposure data (MYL6 ID: eqtl-a-ENSG00000092841; ACSL4 ID: eqtl-a-ENSG00000068366). The subjects of the European population provided these outcome data (see Table 2 for details). Using the “Two Sample MR” program, MR analysis was performed to determine the link between key gene levels (cause) and diseases (effect). The inverse variance–weighted (IVW) approach was utilized. Finally, MR-Egger was used to carry out more sensitivity analysis. Using the “mRnd” online analytical tool^8^, the statistical power of MR estimations was evaluated; a power of more than 80% was considered a noteworthy result.

**Table 2:** List of genome-wide summary association studies (GWAS) in Mendelian randomization (MR) study.

ID	Trait	ncase	group_name	Year	Consortium	Sex	Population	sample_size	ncontrol	nsnp
Exposure										
eqtl-a-ENSG00000092841	ENSG00000092841		Public	2018	NA	Males and females	European	31,684	NA	16,974
eqtl-a-ENSG00000068366	ENSG00000068366		Public	2018	NA	Males and females	European	31,684	NA	10,125
Outcome										
ieu-b-4980	Sepsis	11,643	Public	2021	UK Biobank	Males and females	European	486,484	474,841	12,243,539
ieu-b-4981	Sepsis (28-day death in critical care)	347	Public	2021	UK Biobank	Males and females	European	431,365	431,018	12,243,324
ieu-b-4982	Sepsis (critical care)	1,380	Public	2021	UK Biobank	Males and females	European	431,365	429,985	12,243,372
ieu-b-5086	Sepsis (28-day death)	1896	Public	2021	UK Biobank	Males and females	European	486,484	484,588	12,243,487
ieu-b-5088	Sepsis (under 75)	11,568	Public	2021	UK Biobank	Males and females	European	462,869	451,301	12,243,540

SNP, single-nucleotide polymorphism; NA, not available.

### 2.13 Functional analysis and prediction of SNPs

Through algorithmic predictions and human annotations, the RegulomeDB^9^ is an online resource for interpreting the potential regulatory potential and function of polymorphisms (10). Lower scores in the RegulomeDB indicate stronger evidence that a variation is present in a functional area. SNPs associated with target gene expression and likely to impact binding are indicated by scores 1a through 1 f. SNPs with values of 2a to 2c are those that are likely to have an impact on binding; those with scores of 3a and 3b are less likely to do so; scores of 4, 5, and 6 suggest SNPs that may have little evidence of binding; and a score of 7 indicates that no information is available regarding the function of a particular SNP. Additionally, the RegulomeDB probability score runs from 0 to 1, with values nearer 1 suggesting a high likelihood that this is a regulatory variation (10). Furthermore, using HaploReg v4.1, the functional significance of the non-coding SNPs was found (11). Utilizing variations on haplotype blocks, HaploReg^10^ is a publicly available bioinformatics tool to explore non-coding genomic annotations, such as putative regulatory SNPs at loci for genetic disorders.

VannoPortal^11^ is a comprehensive human variation functional annotation database that integrates a huge amount of variation annotation resources (12). These encompass tissue- and cell-specific gene/epigenome profiles, functional prediction scores at the single-nucleotide level, and a multitude of frequently utilized variation annotation databases. VannoPortal enables the extraction of features for the multi-faceted interpretation of genetic variations, organized into five primary modules: basic variation information, evolutionary insights, disease/trait associations, regulatory potential of variations, and disease pathogenicity. This comprehensive approach facilitates a deeper understanding of genetic variations and their potential impacts on health and disease.

To determine whether SNPs were detrimental, the Sort Intolerant From Tolerant (SIFT) program^12^ was used. Based on the physical properties and sequence homology of amino acids, SIFT can differentiate between the neutral and harmful impacts of amino acid substitutions in SNPs and missense mutations (13). For the purpose of forecasting the structural and functional effects of amino acid alterations, Polymorphism Phenotyping v2 (PolyPhen-2)^13^ is a publicly available web server (14).

### 2.14 Statistical analysis

GraphPad Prism 8.0 or the R program (version 4.2.1)^14^ was used to conduct the statistical analysis. Statistical significance was defined as a *p*-value of less than 0.05 (*p*-value < 0.05; *p-value < 0.01; ***p-value < 0.001), except stated otherwise.

## 3 Results

### 3.1 Identification of DEGs in sepsis and disulfidptosis-related DEGs

**Figure 1:**
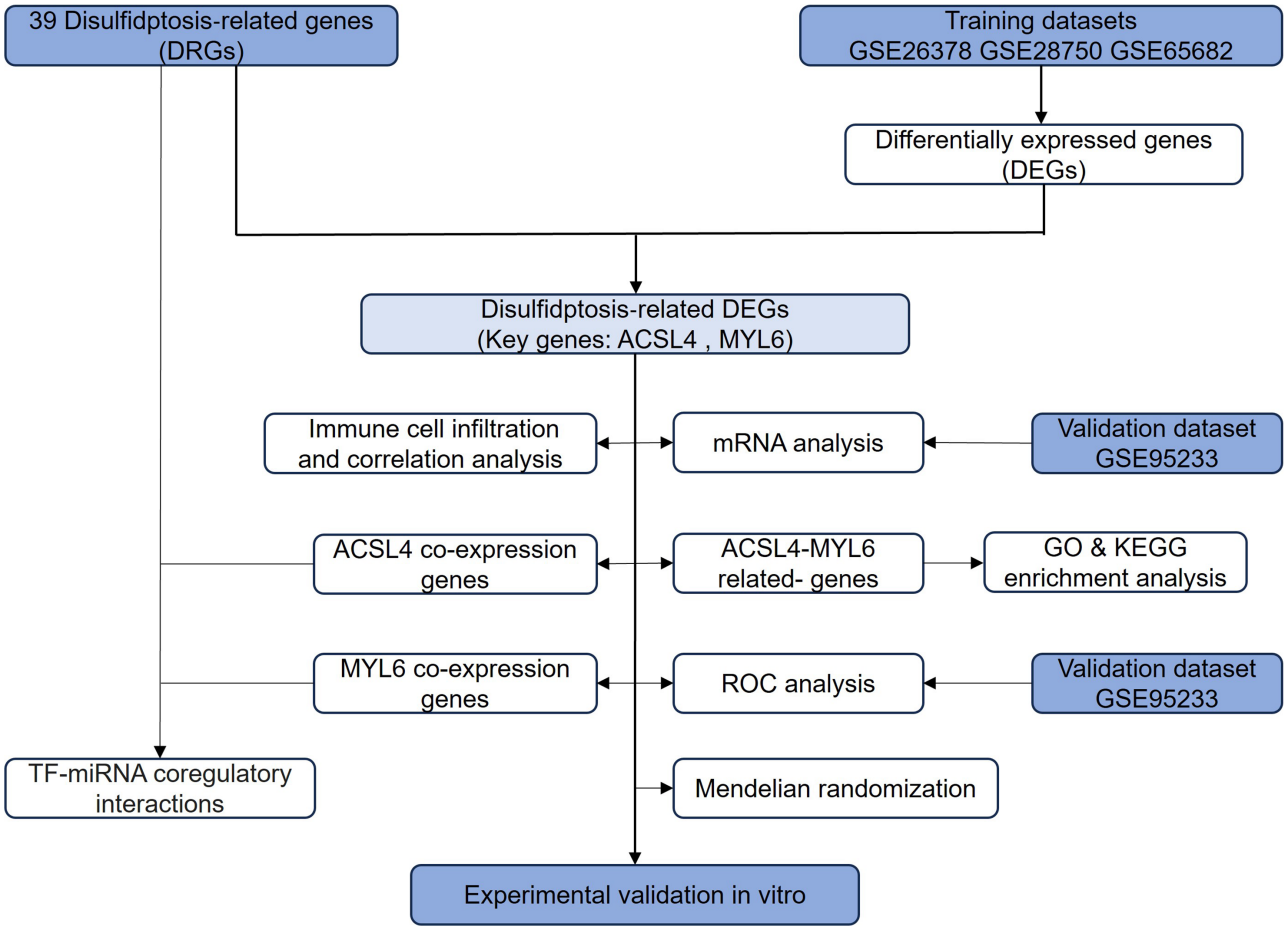
Flowchart of this study. The primary flowchart used in this experimental design of this study. DRGs, disulfidptosis-related genes; DEGs, differentially expressed genes; ROC, receiver operating characteristic; GO, Gene Ontology; KEGG, Kyoto Encyclopedia of Genes and Genomes; miRNA, microRNA.

A flowchart explains the experimental design of this study (Figure 1). We identified two key disulfidptosis-related DEGs in sepsis: ACSL4 and MYL6. Then, we conducted a series of bioinformatic analyses to explore whether two key genes could be targets for sepsis-induced ALI.

**Figure 2:**
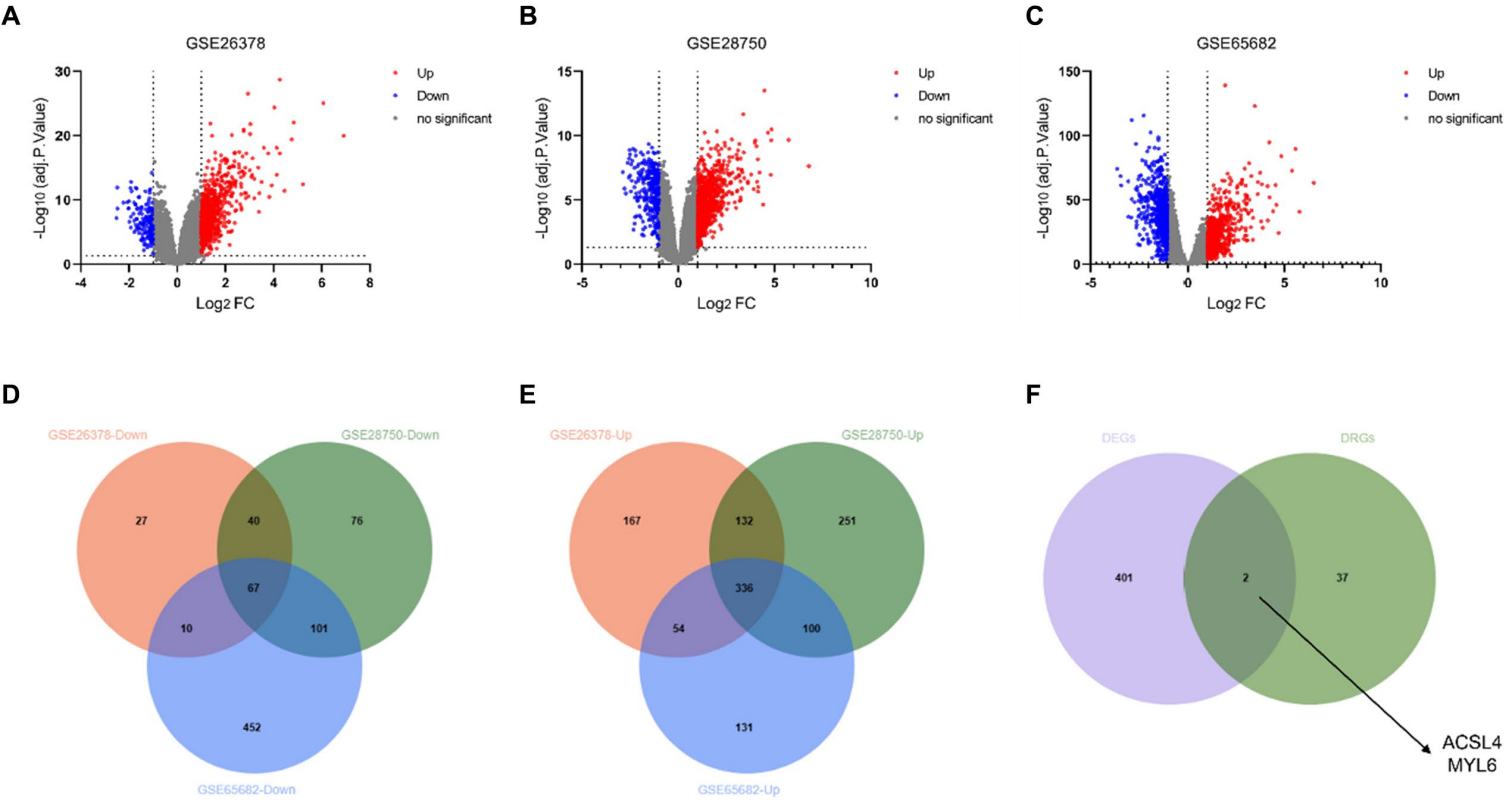
Identification of DEGs and disulfidptosis-related DEGs. Volcano plot of DEGs in the GSE26378 **(A)**, GSE28750 **(B)**, and GSE65682 **(C)** datasets between control groups and sepsis patients. DEGs with downregulated expression are indicated by blue dots, whereas DEGs with upregulated expression are indicated by red dots. Venn diagram of the downregulated genes **(D)** and upregulated genes **(E)** in GSE26378, GSE28750, and GSE65682; **(F)** Venn diagram between DEGs and DRGs.

The DEGs from the three different training datasets were separately identified and visualized by volcano plots (Figures 2A–C). In total, 833 DEGs (689 upregulated and 144 downregulated genes) in the GSE26378 dataset, 1,103 DEGs (819 upregulated and 284 downregulated genes) in the GSE28750 dataset, and 1,251 DEGs (621 upregulated and 630 downregulated genes) in the GSE65682 dataset were shown by the Venn diagram (Figures 2D,E). The common two key genes between 403 DEGs and 39 disulfidptosis-related genes (DRGs) were identified by using the Venn diagram (Figure 2F). ACSL4 and MYL6 as the potential key genes were obtained.

### 3.2 Increased expression of ACSL4 and MYL6 in sepsis groups

**Figure 3:**
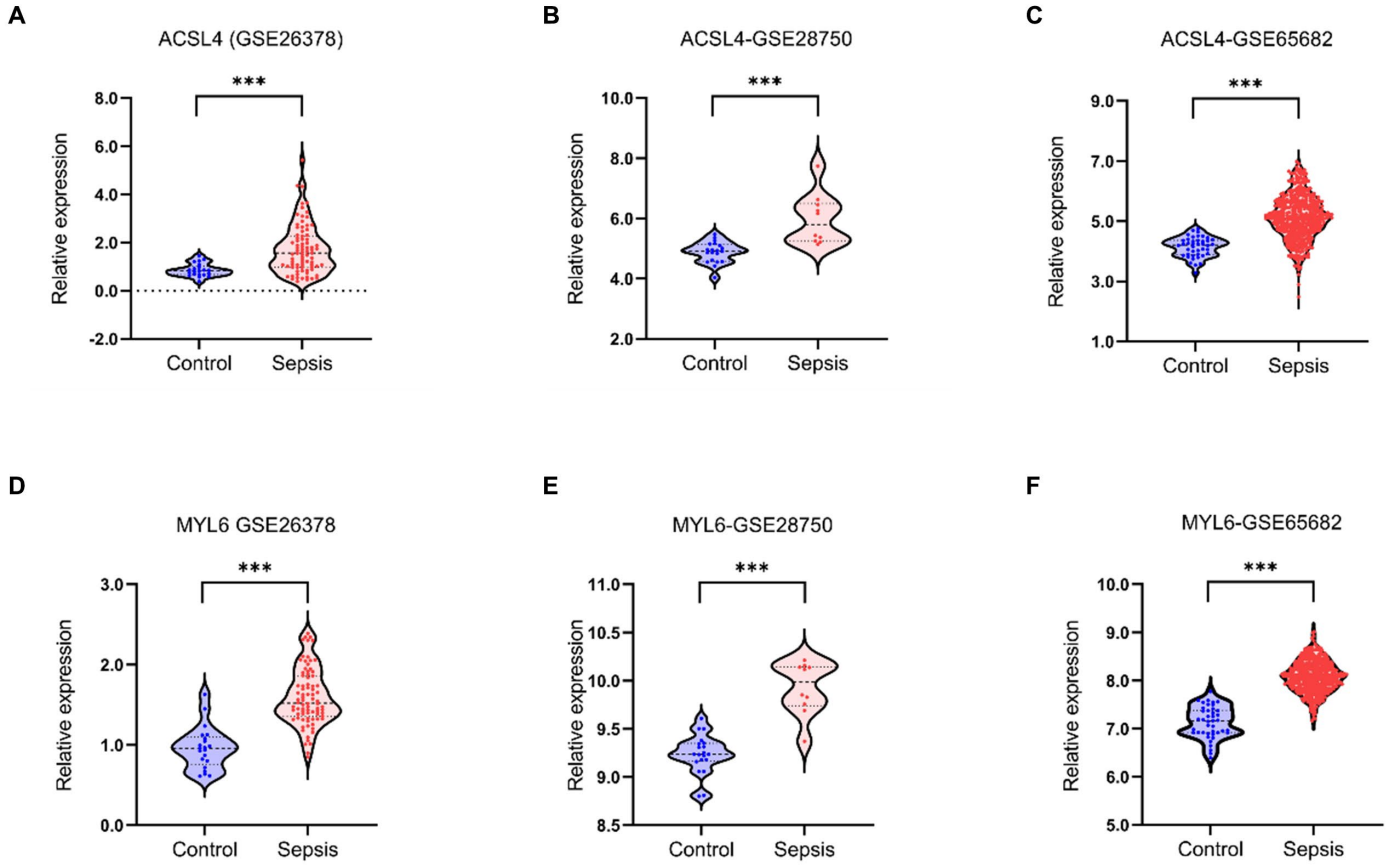
Key genes mRNA levels in training datasets. The mRNA levels of ACSL4 **(A–C)** and MYL6 **(D–F)** in the sepsis group and the control group were detected in the GSE26378, GSE28750, and GSE65682 datasets, respectively.

After identifying ACSL4 and MYL6 as the key genes between DEGs in sepsis and DRGs, we further explored the mRNA levels of two key genes in the GSE26378, GSE28750, and GSE65682 datasets separately. ACSL4 and MYL6 mRNA expressions were significantly upregulated in sepsis groups compared with control groups (*p* < 0.001) (Figure 3).

### 3.3 The correlations between sepsis and immune cell infiltration

**Figure 4:**
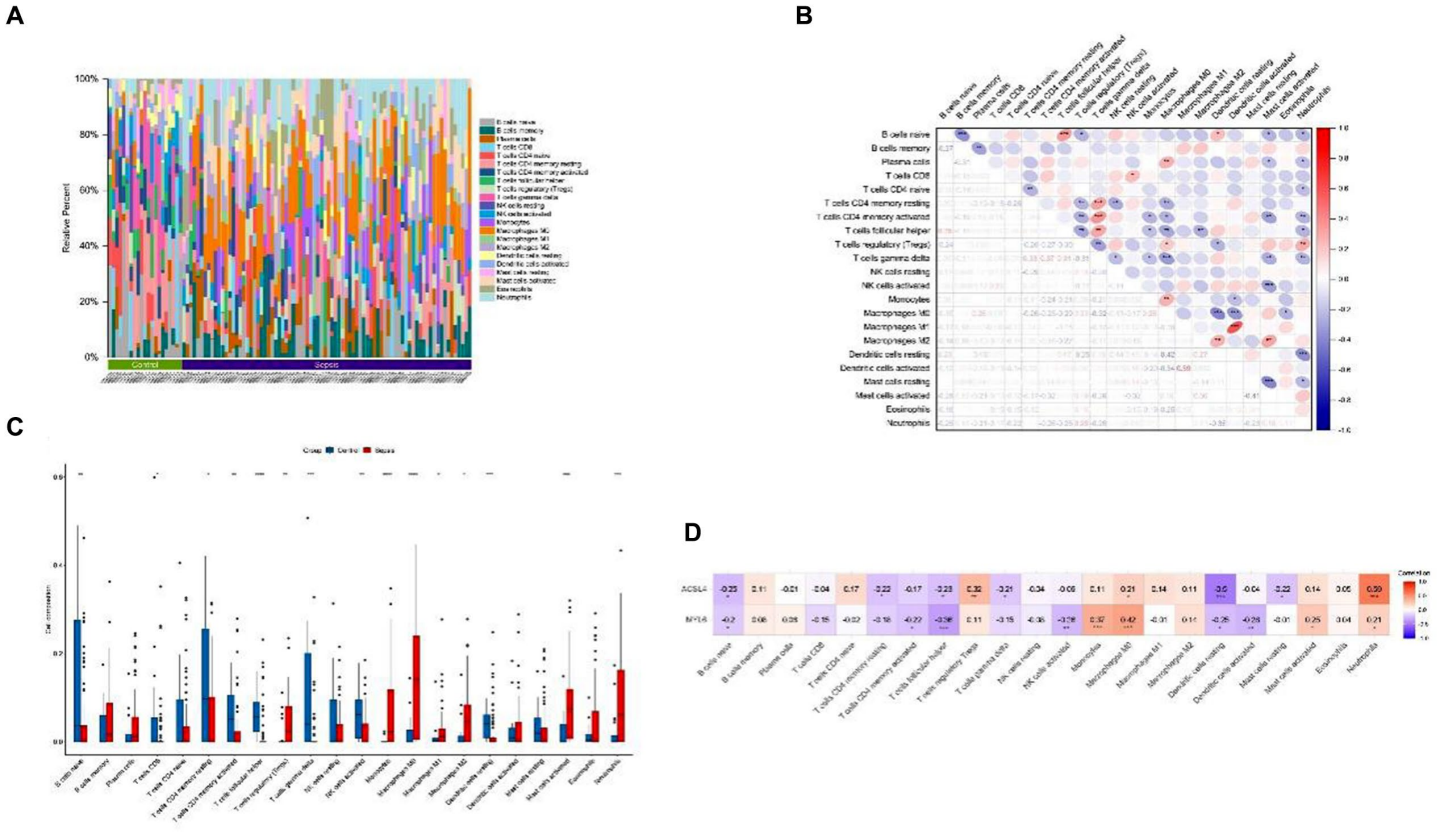
Immune cell infiltration and correlation analysis. **(A)** Relative abundance of 22 types of infiltrating immune cells between the sepsis and the control groups; **(B)** Correlation heatmap of 22 types of immune cells; **(C)** Boxplot of immune cells between the sepsis and the control groups; **(D)** Heatmap of correlations between immune cells and key genes. *p < 0.05, **p < 0.01, ***p < 0.001.

To investigate the potential association between sepsis and immunity, we used the CIBERSORT algorithm to quantify the relative abundance of 22 types of infiltrating immune cells in both sepsis and control groups (Figure 4A). The correlation of 22 kinds of immune cells was shown by heatmap (Figure 4B). The color depth of the box indicates the correlation intensity between sepsis and immune cells: red represents a positive correlation, blue represents a negative correlation, and darker color represents stronger connection. The lower triangular represents the correlation coefficient, and the upper triangular shows the significant mark. The boxplot revealed significant differences in 15 of 22 types of immune cells between sepsis and control groups (Figure 4C). Above all, these results indicated that immunological dysfunction plays a potentially significant role in the pathogenesis and progression of sepsis. Subsequently, the heatmap was used to further analyze the potential correlations between the key genes and infiltrating immune cells, we found that ACLS4 correlated with regulatory T cells (Tregs) (*r* = 0.32), macrophages M0 (*r* = 0.21), neutrophils (*r* = 0.59) showing a positive correlation, and with B cells native (*r* = −0.25), T-cell CD4 memory resting (*r* = −0.22), T–cell follicular helper (*r* = −0.23), T-cell gamma delta (*r* = −0.21), resting of dendritic cells (*r* = −0.5), mast cell resting (*r* = −0.22) showing a negative correlation. For another key gene MYL6, ACLS4 positively correlated with monocytes (*r* = 0.37), macrophages M0 (*r* = 0.42), mast cell activated (*r* = 0.25), neutrophils (*r* = 0.21), and a negative correlation with B cells native (*r* = −0.2), T-cell CD4 memory activated (*r* = −0.22), T-cell follicular helper (−0.36), NK cell activated (*r* = −0.26), dendritic cells resting (*r* = −0.25), and dendritic cells activated (*r* = −0.28) (Figure 4D). The results indicated that both two key genes ACLS4 and MYL6 might contribute to the immune infiltration status of patients with sepsis.

### 3.4 PPI network and functional analysis for the key genes and their related genes

**Figure 5:**
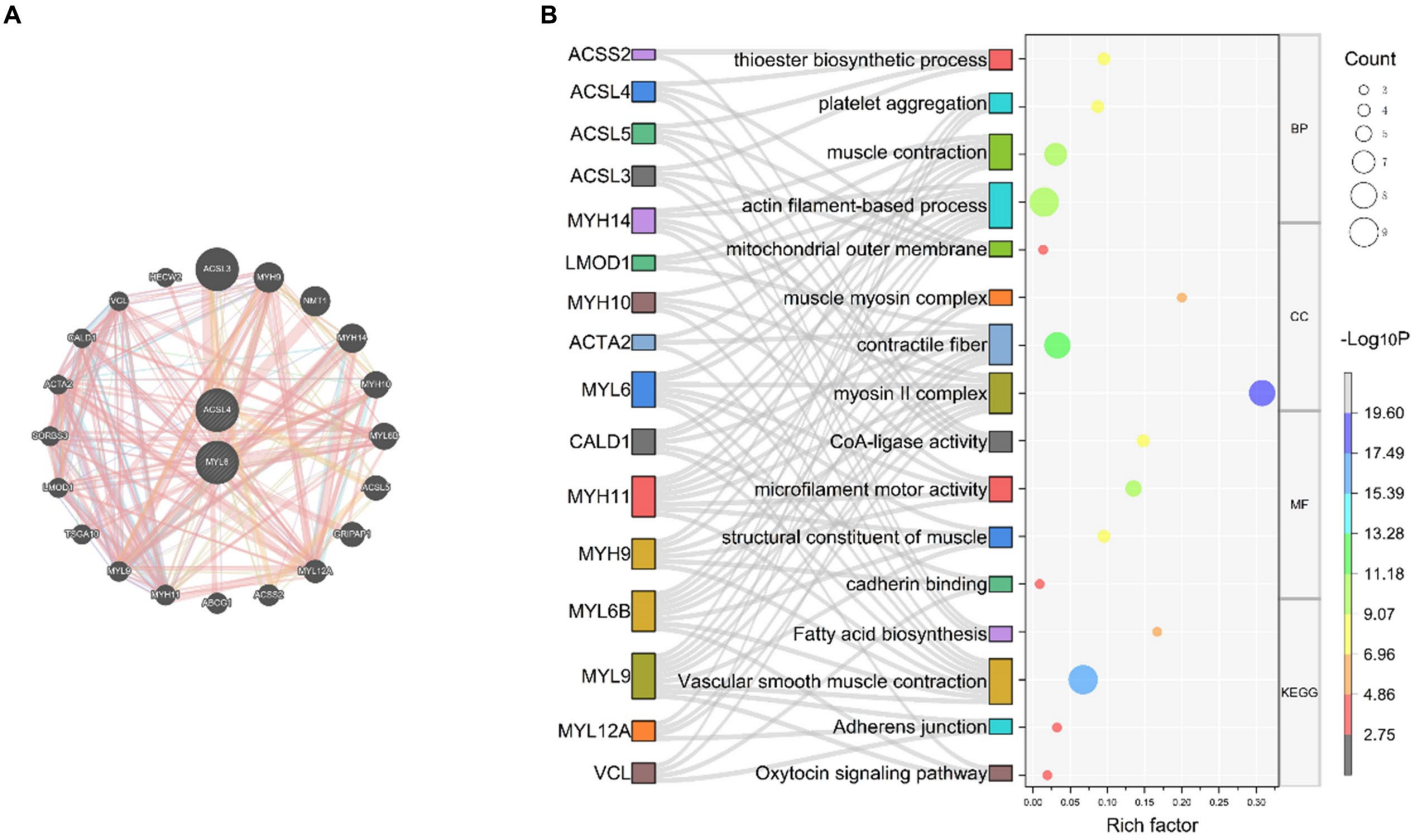
PPI network and function prediction for the key genes and their related genes **(A)** PPI network of ACSL4 and MYL6; **(B)** GO enrichment and KEGG pathways analyses of related genes using the Sankey bubble chart. BP, Biological process; CC, cellular component; MF, molecular function; KEGG, Kyoto Encyclopedia of Genes and Genomes.

Based on the results of the above analysis, we further explored the biological link between key genes and sepsis. To build PPI networks for ACSL4 and MYL6, we used the GeneMANIA database, which allows us to estimate the function of particular genes and gene sets (Figure 5A). In this section, a total of 22 related genes were evaluated and arranged according to correlation: ACSL4, MYL6, ACSL3, MYH9, NMT1, MYH14, MYH10, MYL6B, A5CSL5, GRIPAP1, MYL12A, ACSS2, ABCG1, MYH11, MYL9, TSGA10, LMOD1, SORBS3, ACTA2, CALD1, VCL, and HECW2.

In order to investigate the biological functions and pathways of the key genes, GO and KEGG enrichment analyses were performed (Figure 5B). According to GO enrichment analysis results, it proved that related genes of key genes were significantly enriched in actin filament-based process, muscle contraction, thioester biosynthetic process, and platelet aggregation at biological process (BP) levels; myosin II complex, contractile fiber, mitochondrial outer membrane, and muscle myosin complex at cellular component (CC) levels; microfilament motor activity, CoA-ligase activity, structural constituent of muscle, and cadherin binding at molecular function (MF) levels. Related genes of key genes were significantly enriched in the following KEGG pathways, including vascular smooth muscle contraction, fatty acid biosynthesis, adherens junction, and oxytocin signaling pathway (Figure 5B).

### 3.5 Identification and network analysis of key genes co-expression genes associated with sepsis

**Figure 6:**
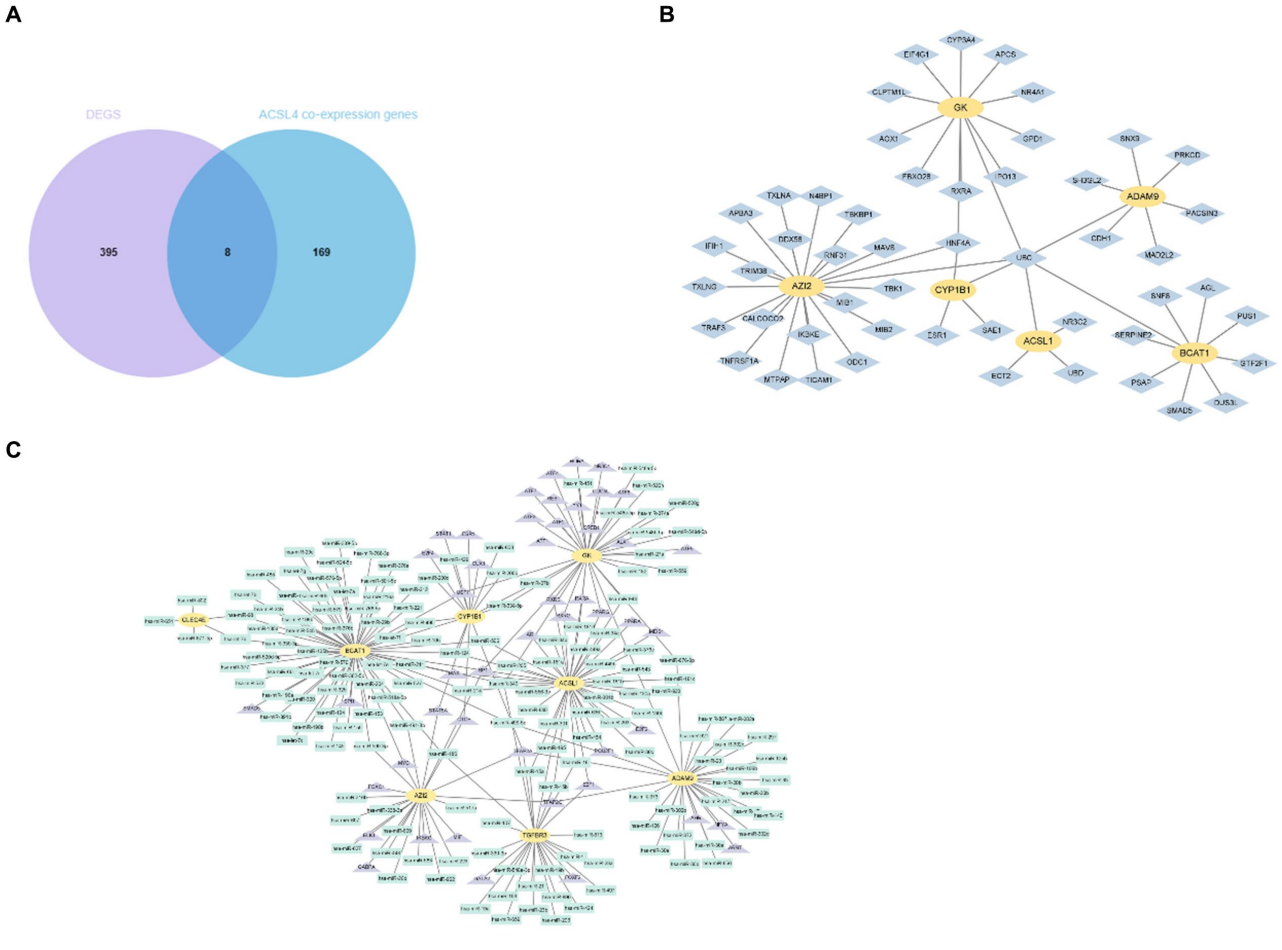
Identification and network analysis of overlapping genes between ACSL4 co-expression genes and DEGs. **(A)** Venn diagram showing the overlapping genes between ACSL4 co-expression genes and DEGs in sepsis; **(B)** PPI network of the discovered overlapping genes; **(C)** TF-miRNA coregulatory interactions of discovered overlapping genes.

To further investigate the potential relationship and the underlying mechanisms between key genes and sepsis, we acquired ACSL4 and MYL6 co-expression genes from the Coexpedia dataset and then intersected with 403 DEGs in sepsis. The Venn diagram shows that a total of eight overlapping genes were discovered between ACSL4 co-expression genes and sepsis, including GK, CLEC4E, ACSL1, TGFBR3, ADAM9, AZI2, BCAT1, and CYP1B1 (Figure 6A). Then, we determined interactions among the overlapping genes through the PPI network, which can regulate various biological processes (Figure 6B).

**Figure 7:**
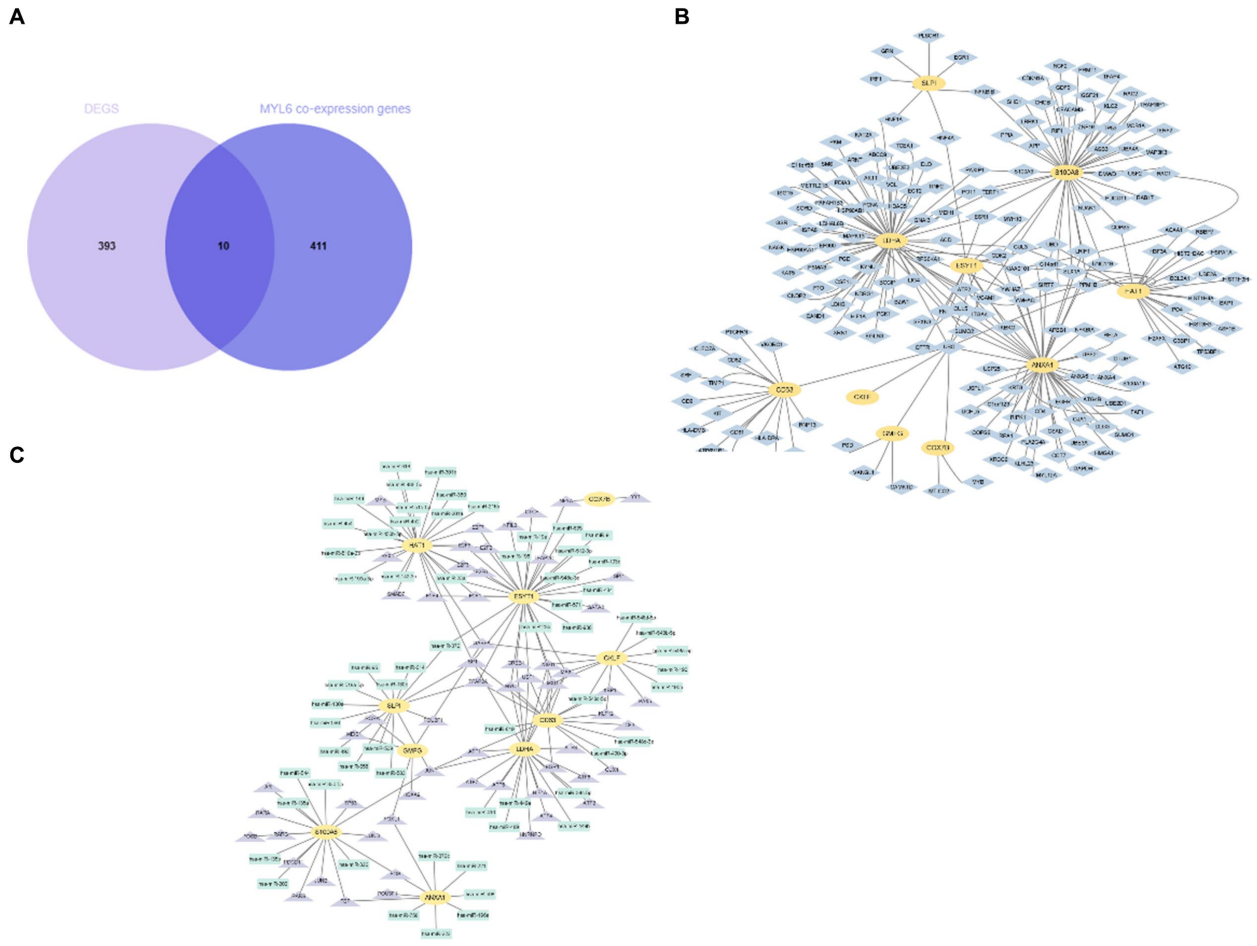
Identification and network analysis of overlapping genes between MYL6 co-expression genes and DEGs. **(A)** Venn diagram showing the overlapping genes between MYL6 co-expression genes and DEGs in sepsis; **(B)** PPI network of the discovered overlapping genes; **(C)** TF–miRNA coregulatory interactions of discovered overlapping genes.

As we know, the transcript factor (TF) regulates transcription by binding promoter region, whereas the miRNA regulates post-transcriptional gene expression. The TF–miRNA coregulatory interactions can affect gene expression and are closely related to the occurrence and prognosis of many complex diseases (15). Hence, the TF–miRNA coregulatory interactions composed of these genes were used to search the latent genetic regulation between ACSL4 and sepsis (Figure 6C). It was found that three TFs (SP1, TFAP2A, and TFAP2C) and three miRNAs (hsa-miR-340, hsa-miR-543, and hsa-miR-124) were most closely related in this network. Then, we analyzed MYL6 co-expression genes associated with sepsis in the same way. A total of 10 overlapping genes were found in the Venn diagram, including ESYT1, ANXA1, CKLF, CD63, GMFG, HAT1, LDHA, SLPI, COX7B, S100A8 (Figure 7A). To contextualize the shared biochemical function and transcriptomic signature, we still constructed a comprehensive network of PPI network and TF–miRNA coregulatory interactions of discovered overlapping genes (Figures 7B,C). Among the miRNAs targeting multiple shared genes, hsa-miR-370, hsa-miR-548c-5p, and hsa-miR-23a sprang out as the signatures with great connections.

### 3.6 Potential diagnostic value of key genes

**Figure 8:**
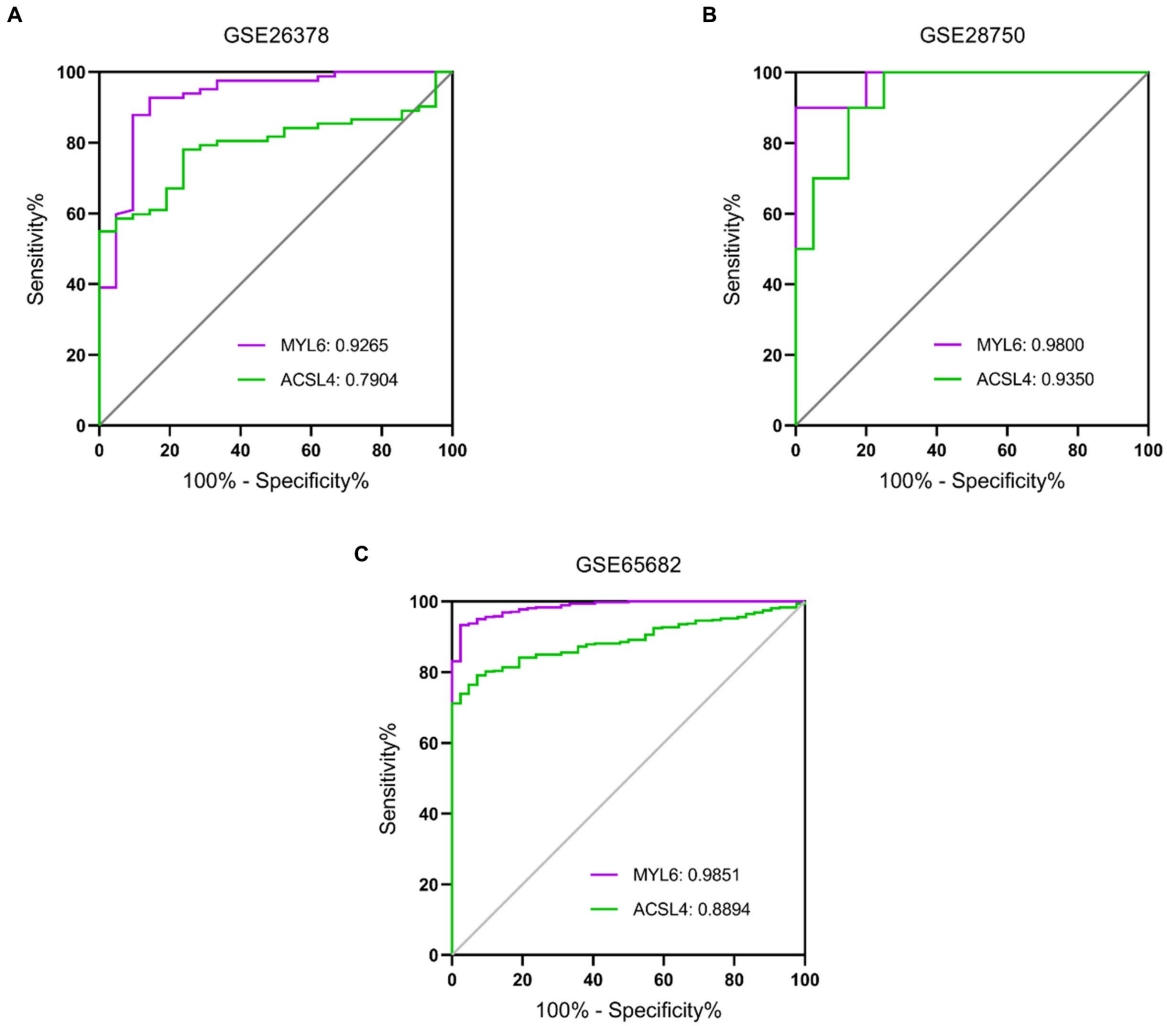
Performance of the key genes in the diagnosis of sepsis in the training datasets. ROC curves of the two key genes in GSE26378 **(A)**, GSE28750 **(B)**, and GSE65682 **(C)** datasets. The AUC of MYL6 and ACSL4 in different datasets is shown.

To further evaluate the potential diagnostic value of ACSL4 and MYL6 in sepsis, receiver operating characteristic (ROC) curve analyses were plotted based on the two gene expressions in different training datasets (Figure 8). AUC for MYL6 and ACSL4 in the GSE26378 dataset were 0.9265 and 0.7904, respectively (MYL6 cutoff: 1.142%; sensitivity: 92.68%; specificity: 85.71% vs. ACSL4 cutoff: 1.466%; sensitivity: 54.88%; specificity: 100%) (Figure 8A). In the GSE28750 dataset, the AUCs for MYL6 and ACSL4 were 0.9800 and 0.9350, respectively (MYL6 cutoff: 9.649%; sensitivity: 90%; specificity: 100% vs. ACSL4 cutoff: 5.193%; sensitivity: 90%; accuracy: 85%) (Figure 8B). With respect to MYL6 and ACSL4, the AUC in the GSE65682 dataset was 0.9851 and 0.8894, respectively (MYL6 cutoff: 7.594%; sensitivity: 93.32%; specificity: 97.62% vs. ACSL4 cutoff: 4.555%; sensitivity: 79.12%; specificity: 92.86%) (Figure 8C). These findings demonstrated that ACSL4 and MYL6, particularly MYL6, are good discriminators for sepsis patients.

**Figure 9:**
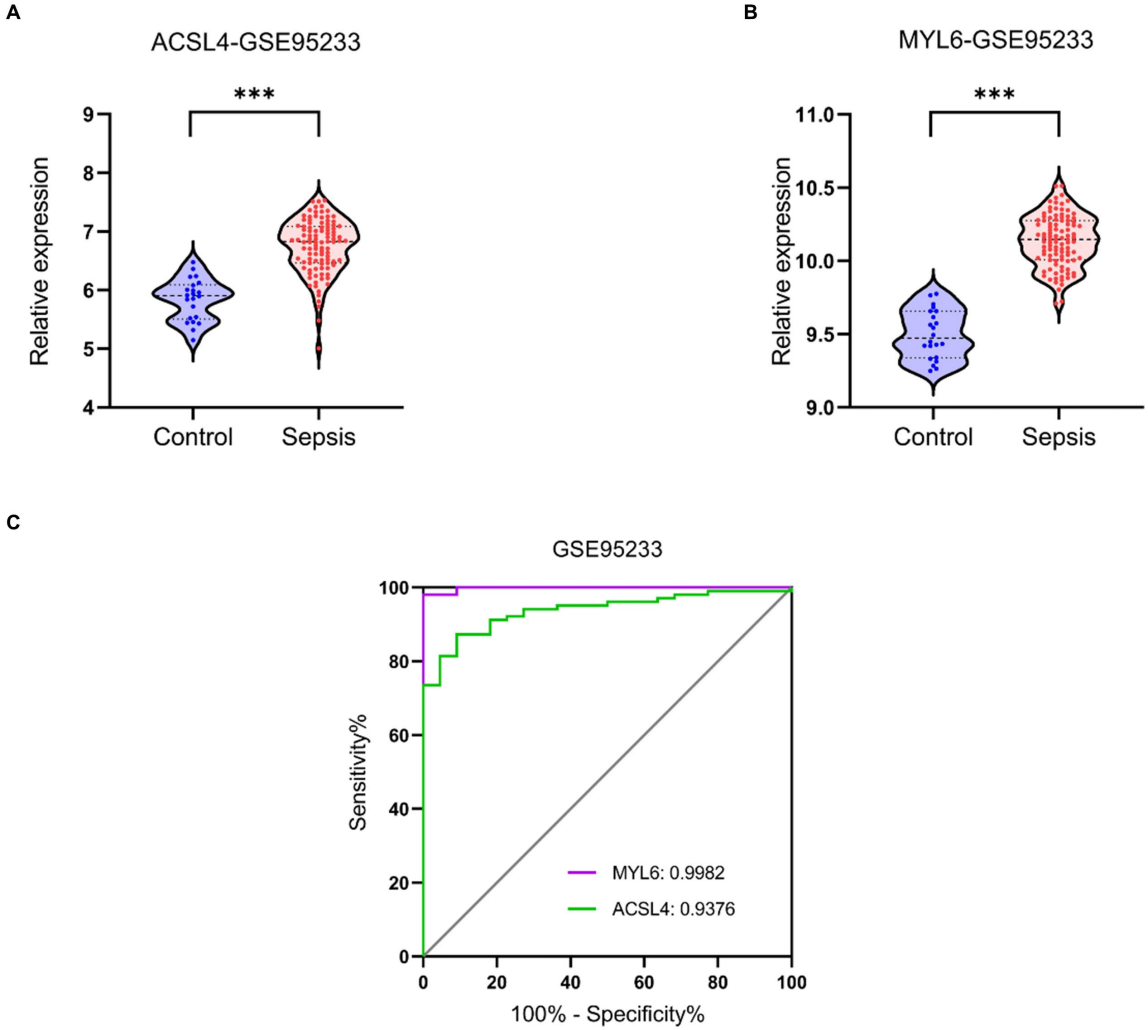
Key genes mRNA levels and ROC analysis in the validation dataset. **(A,B)** ACSL4 and MYL6 mRNA expressions in sepsis and control groups were detected in the GSE95233 dataset, respectively; **(C)** ROC curves of the two key genes in the GSE95233 dataset.

Validation datasets were used to verify the accuracy of the conclusion of the three training datasets. The expressions of ACSL4 and MYL6 mRNA in the GSE95233 dataset were significantly increased in the sepsis group compared with the control group (*p* < 0.001) (Figures 9A,B). The AUCs of MYL6 and ACSL4 were all greater than 0.9 (Figure 9C). Furthermore, the diagnostic sensitivity and specificity of MYL6 were 98.04% and 100%, when the cutoff value was 9.789%, according to ROC curve analysis in the GSE95233 dataset. Moreover, the cutoff value, sensitivity, and specificity of ACSL4 were 6.238%, 87.25%, and 90.91%, respectively (Figure 9C). Given the above, the accuracy of the above results of training datasets was confirmed.

### 3.7 The levels of ACSL4 and MYL6 upregulated in sepsis-induced ALI models

**Figure 10:**
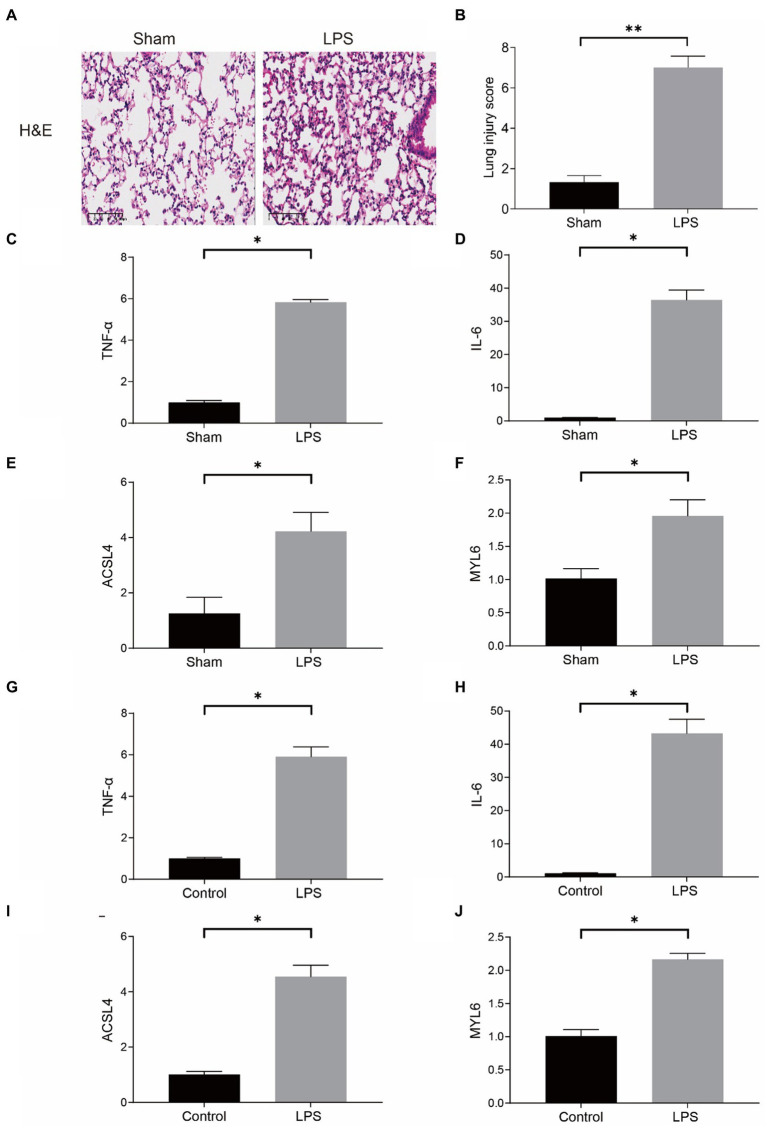
mRNA levels of key genes in the mouse model of sepsis-induced ALI and in the LPS-treated RAW264.7. **(A)** HE staining in the Sham group and the LPS group; **(B)** Lung injury score in the Sham group and the LPS group; mRNA levels of TNF-α **(C)**, IL-6 **(D)**, ACSL4 **(E)**, and MYL6 **(F)** in the mouse model; The mRNA levels of TNF-α **(G)**, IL-6 **(H)**, ACSL4 **(I)**, and MYL6 **(J)** in the cell model.

Our conjecture was further demonstrated in LPS-induced sepsis models. In the mouse model, the results of HE staining indicated that the pulmonary architecture of control mice was normal in shape and clear in structure. Compared to the Sham group, pulmonary tissue with LPS challenges were significantly damaged, involving alveolar disarray and alveolar septa thickened, as well as alveolar congestion was more obvious in the LPS group (Figure 10A). We could also observe that the lung injury score in the LPS group was much higher than the Sham group (Figure 10B). Furthermore, the results of qPCR showed that the mRNA levels of proinflammatory cytokines, such as tumor necrosis factor-α (TNF-α), and interleukin-6 (IL-6), were substantially upregulated in the LPS group (Figures 10C,D). The above results proved that the model of LPS-induced ALI in mice was successful. More importantly, our qPCR results indicated that the mRNA levels of ASCL4 and MYL6 in the pulmonary tissue of mice in the LPS group were increased relative to the Sham group (Figures 10E,F).

In the meantime, RAW264.7 cells were used for determining the mRNA levels of ACSL4 and MYL6 in a cell model *in vitro*. The contents of TNF-α and IL-6 in the LPS group were significantly higher than those in the control group, which indicated that LPS successfully induced inflammation in cells (Figures 10G,H). As expected, LPS increased the levels of ACSL4 and MYL6 mRNA in RAW264.7 cells (Figures 10I,J).

Both *in vivo* and *in vitro* results were consistent, indicating higher ACSL4 and MYL6 mRNA levels in LPS-induced septic mice and cells, suggesting that ACSL4 and MYL6 may be potential therapeutic targets for sepsis-induced ALI.

### 3.8 The level of disulfidptosis was upregulated in sepsis-induced ALI models

**Figure 11:**
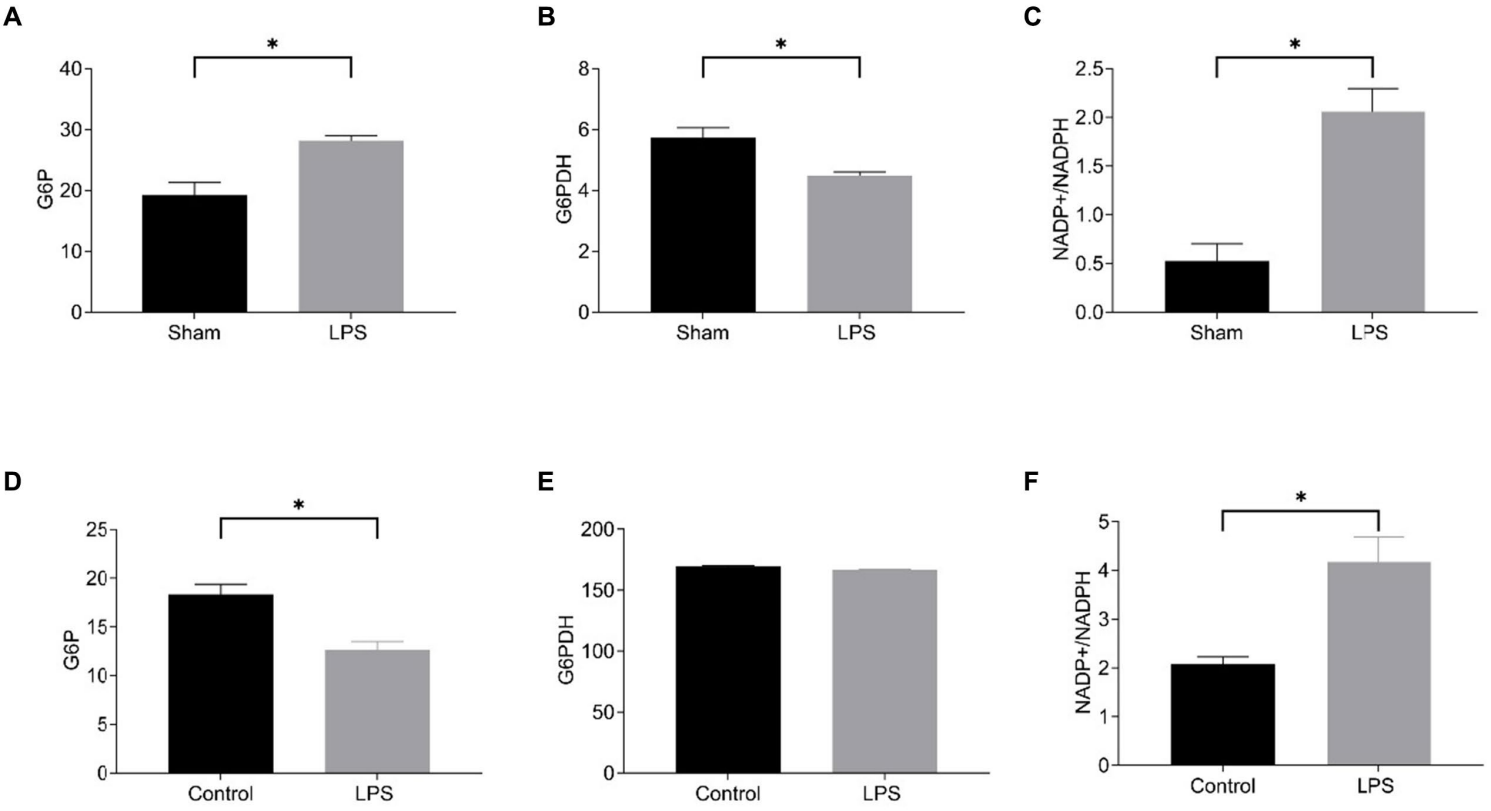
Level of disulfidptosis in the mouse model of sepsis-induced ALI and in the LPS-treated RAW264.7. The G6P content **(A)**, G6PDH activity **(B)**, and NADP+/NADPH ratio **(C)** in the mouse model; The G6P content **(D)**, G6PDH activity **(E)**, NADP+/NADPH ratio **(F)** in the cell model.

Then, we detected disulfidptosis-related markers in LPS-induced lung tissue and RAW264.7. We chose to measure the NADP+/NADPH ratio, glucose-6-phosphate (G6P) content, and glucose 6-phosphate dehydrogenase (G6PDH) activity. G6P undergoes oxidation under the action of G6PDH to generate 6-phosphogluconate (6-PG), during which NADP+ is reduced to NADPH. Regardless of whether NADPH generation decreases or consumption increases, it will lead to insufficient NADPH content, which cannot meet the process of cysteine reduction to cysteine, and induce disulfide cross-linking and cytoskeletal contraction of actin cytoskeletal proteins, ultimately inducing disulfidptosis (6). The results showed that in LPS-induced sepsis lung tissue, compared with the Sham group, G6P content was increased, G6PDH activity was decreased, and NADP+/NADPH ratio was increased in the LPS group (Figures 11A–C). In RAW264.7 cells, compared with the control group, G6P content was decreased in the LPS group, the activity of G6PDH was not significantly changed, and the NADP+/NADPH ratio was increased (Figures 11D–F). Although G6P and G6PDH showed different trends in LPS-induced RAW264.7 and lung tissue, they both resulted in an elevated NADP+/NADPH ratio, indicating enhanced disulfidptosis. The results showed disulfidptosis may play a crucial role in sepsis-induced lung injury.

### 3.9 Causal association between ACSL4/MYL6 and sepsis

In the study, the causal link between sepsis and ACSL4/MYL6 was further investigated. With an OR of 2.169 (95% CI = 1.259–3.736, *p* = 0.005), we discovered a strong association between the MYL6 gene and the risk of sepsis (a 28-day death in critical care) using the IVW technique (see Table 3; Supplementary Figure S1 for details). Using the IVW approach, power was more than 80% for both MYL6 on sepsis (a 28-day death in critical care) (Table 3). On the other hand, there was no discernible impact of ACSL4 on sepsis (for further information, see Table 3; Supplementary Figure S1). The absence of bias in the causative effect was further confirmed by the intercept of the MR-Egger regression and IVW, which did not show horizontal pleiotropy (see Table 4 for details). Supplementary Figure S1 displays scatter plots of valuable MR predictions. Supplementary Figures S2, S3 exhibit the leave-one-out sensitivity analyses and forest plots of all suggestively relevant regulators. Supplementary Figure S4 presents a funnel plot illustration.

**Table 3:** Two-sample MR analysis results of MYL6/ACSL4 and sepsis.

Exposure	Outcome	Method	NSNP	pval	OR	OR_95%LCI	OR_95%UCI	Statistical power
MYL6	Sepsis	IVW	10	0.419	0.962	0.876	1.057	0.25
	Sepsis (28-day death in critical care)	IVW	10	0.005	2.169	1.259	3.736	1.00
	Sepsis (critical care)	IVW	10	0.102	1.252	0.956	1.640	1.00
	Sepsis (28-day death)	IVW	10	0.240	1.148	0.912	1.444	0.98
	Sepsis (under 75)	IVW	10	0.814	1.012	0.915	1.120	0.07
ACSL4	Sepsis	IVW	7	0.470	1.113	0.833	1.488	0.93
	Sepsis (28-day death in critical care)	IVW	7	0.066	3.314	0.926	11.863	1.00
	Sepsis (critical care)	IVW	7	0.271	1.488	0.733	3.023	1.00
	Sepsis (28-day death)	IVW	7	0.551	1.171	0.697	1.965	1.00
	Sepsis (under 75)	IVW	7	0.851	0.970	0.703	1.338	0.16

NSNP, number of single-nucleotide polymorphisms number; OR, odds ratio; CI, confidence interval; UCI, upper limit of the confidence interval; LCI, lower limit of the confidence interval.

**Table 4:** Tests for horizontal pleiotropy and heterogeneity in the MR analysis.

Exposure	Outcome	Horizontal pleiotropy test			Heterogeneity test			
		egger_intercept	se	pval	Method	Q	Q_df	Q_pval
MYL6	Sepsis	−0.00461	0.0120	0.710	MR-Egger	8.103	8	0.423
					IVW	8.254	9	0.509
	Sepsis (28-day death in critical care)	−0.129	0.0685	0.0957	MR-Egger	3.168	8	0.923
					IVW	6.733	9	0.665
	Sepsis (critical care)	−0.0211	0.0341	0.553	MR-Egger	6.775	8	0.561
					IVW	7.159	9	0.621
	Sepsis (28-day death)	−0.00959	0.0290	0.750	MR-Egger	6.567	8	0.584
					IVW	6.676	9	0.671
	Sepsis (under 75)	0.00637	0.0133	0.646	MR-Egger	10.535	8	0.229
					IVW	10.834	9	0.287
ACSL4	Sepsis	0.0266	0.0264	0.361	MR-Egger	9.3610	5	0.096
					IVW	11.254	6	0.0808
	Sepsis (28-day death in critical care)	0.226	0.111	0.0974	MR-Egger	2.428	5	0.787
					IVW	6.574	6	0.362
	Sepsis (critical care)	0.0402	0.0685	0.582	MR-Egger	7.602	5	0.180
					IVW	8.127	6	0.229
	Sepsis (28-day death)	0.00156	0.0472	0.975	MR-Egger	2.087	5	0.837
					IVW	2.088	6	0.911
	Sepsis (under 75)	0.0230	0.0304	0.483	MR-Egger	12.951	5	0.0238
					IVW	14.437	6	0.0251

MR, Mendelian randomization; IVW, inverse variance weighted.

## 4 Discussion

Sepsis is one of the most common complications in the intensive care unit (ICU) (16). Due to its complex pathogenesis and rapid disease progression, its therapeutic methods were more difficult and the case fatality rate was higher than other diseases. Sepsis-induced ALI can further evolve into acute respiratory distress syndrome (ARDS), which is characterized by a sudden aggravation of non-cardiogenic pulmonary edema, severe hypoxemia, is the fatal consequence of severe sepsis (2). Currently, the damage of vascular endothelial cells, alveolar epithelial cells, and epigenetics caused by inflammatory response, immunologic function derangement, destruction of intercellular connection and integrity, and special cell death pathway were the main pathogenic mechanisms of sepsis-induced ALI (2, 17–19). However, there is still a lack of effective targets for treating sepsis-induced ALI.

Disulfidptosis is the novel type of cell death pathway. It is primarily caused by the accumulation of disulfide bonds, thus leading to the collapse of the cytoskeleton and subsequent cell death (6), which is closely related to disease progression. However, the specific mechanisms of disulfidptosis and its regulatory role in various diseases, as well as the underlying pathways, have not been further studied. Here, we attempt to elucidate the role of disulfidptosis-related genes in sepsis-induced ALI by linking disulfidptosis-related genes to the pathogenesis of sepsis-induced ALI and identifying potential key genes through bioinformatics analysis to explore potential therapeutic targets.

To investigate the potential role of disulfidptosis in sepsis-induced ALI, first we used GEO2R online analysis software to identify DEGs and then intersected with DRGs to obtain the key genes: ACSL4 and MYL6. ACSL4 is one member of the long-chain acyl-CoA synthetase family (ACSLs), which is an important isoenzyme of polyunsaturated fatty acid (PUFA) metabolism. ACSL4 catalyzes the connection between long-chain PUFAs and CoA and participates in the regulation of arachidonic acid, then promoting phospholipid remodeling by adding non-esterified fatty acid (20). Bone marrow cell-specific ACSL4 deficiency can alleviate inflammation by remodeling phospholipids and reducing proinflammatory lipid mediators derived from arachidonic acid (21). MYL6 is an essential non-phosphorylated alkali light chains of myosin that regulates the cell shape alteration and migration by interacting with F-actin (22). The study has revealed that anti-MYL6 antibody can decrease the disease activity of microscopic polyangiitis by damaged actin rearrangement necessary for neutrophil extracellular trap formation (22). The current research showed that MYL6 plays an important role in the form of cytoskeletal structures and it may be beneficial to stabilize the myosin structure (23). Based on many present studies, the influence of ACSL4 and MYL6 on cell structure and movement is closely related to the occurrence and development of cancer, inflammation, ischemia–reperfusion, and other diseases.

Consistent with previous research findings, our investigation demonstrated a substantial upregulation of ACSL4 mRNA in sepsis-induced ALI (24, 25). Furthermore, our study showed for the first time that the mRNA levels of MYL6 increase in sepsis, consistent with ACSL4 results. This portion of the data pointed to a possible connection between key genes and the progression of sepsis. However, further research into the underlying mechanism is still necessary. As we know, immune cells such as neutrophils, macrophages, monocytes, and dendritic cells (DCs) are crucial in the pathogenesis of infection-related ALI and its outcome (19). Among those immune cells, lung immune cells at homeostasis were mainly composed of pulmonary macrophages accounting for 90–95% (26). The main function of macrophage is to eliminate pathogens by phagocytosis. Macrophages M0 are differentiated from monocytes induced by M-colony-stimulating factor (CSF), and then, they are divided into two categories according to different stimulating factors, namely M1 type macrophages and M2 type macrophages (27). The former shows proinflammatory role, whereas the latter plays an anti-inflammatory role (28). Neutrophils as the other immune cells to eliminate invading bacteria, which were considered the main defense at the early stage of infection (29). Therefore, restoring the function of immune cells was vital for sepsis and secondary organ damage. The significant differences in 15 of 22 types of immune cells between the sepsis and control samples were revealed by the CIBERSORT algorithm, suggesting the potential role of immune cells in the development of sepsis. The heat map showed that both of ACSL4 and MYL6 had a positive correlation with infiltrating immune cells; in particular, macrophages M0 and neutrophils were much stronger than others. In addition, both *in vivo* and *in vitro* results have shown that in LPS-induced septic mice and cells, much higher ACSL4 and MYL6 mRNA levels were displayed, suggesting ACSL4 and MYL6 may possess the capacity to influence sepsis by inflammation response and immune infiltration. Wang and colleagues revealed that inhibition of ACSL4 can alleviate inflammatory response by reducing the infiltration of macrophages and neutrophils, then protecting mice from I/R- and FA-induced AKI (30). Those results verified our conjecture about the potential connection between ACSL4 and sepsis.

To further understand the key genes biological characteristics, we build PPI networks for ACSL4 and MYL6, and those related genes were carried out GO and KEGG analyses. Kai et al.’s study discovered that in mice with an LPS-mediated ALI model, the expression of the ACSL4 protein increased in lesion tissues, indicating there is a potential function for ACSL4 in sepsis-related acute lung injury (24). ACSS2 encodes acetyl-CoA synthetase 2 and is involved in the generation of acetyl-CoA, which is an important intermediate in a variety of metabolic pathways. In sepsis, acetyl-CoA levels may affect cellular energy metabolism and inflammatory signaling pathways. Moreover, studies have shown that the renal epithelial cells of mice with LPS-induced AKI were shown to have considerably higher expression of ACSS2. This suggests that ACSS2 may play an important role in sepsis (31). ABCG1, encoding ATP-binding cassette transporter G1, was involved in cholesterol and phospholipids of transshipment. In sepsis, metabolism and transport of these substances may influence the integrity of the cell membrane and inflammatory response. Studies have found that, in LPS-induced endotoxemia in mice, LPS inhibit ABCG1 expression and promote the development of inflammation (32). However, association of other genes with sepsis have not yet been reported. The GO analysis revealed that related genes of key genes were mainly related to actin filament-based process, muscle contraction, myosin II complex, contractile fiber, microfilament motor activity, structural constituent of muscle. Previous research has demonstrated that numerous molecular pathways in sepsis can regulate and impact actin dynamics, leading to aberrant vascular permeability and endothelial barrier function. The above alters the integrity of endothelial cells and ultimately contributes to the development and progression of sepsis (33). In addition, Kristen T. Crowell and Charles H. Lang found that the reduction of 50 to 90% of thin filament (such as tropomyosin and a-sarcomeric actin), thick filament (myosin heavy and myosin light chains), Z-disk (a-actinin-3), and M-band (myomesin-2) proteins contributes to the intrinsic functional defects of muscle contraction during the sepsis recovery phase (34). While in the phase of disulfidptosis, the actin network collapses and disulfide proteins accumulate as a result of excessive disulfide bond formation in actin cytoskeleton proteins (9). These conclusions are roughly consistent with our research results, suggesting that the key genes and their related genes may affect the occurrence and development of sepsis by affecting the stability of cells and the integrity of the cytoskeleton. KEGG analysis suggested that the vascular smooth muscle contraction and fatty acid biosynthesis were significantly enriched. Although vascular smooth muscle contraction and fatty acid biosynthesis are primarily associated with cardiovascular diseases such as atherosclerosis, central nervous system (CNS) diseases, and cancer (35), there is an increasing evidence to suggest its involvement in sepsis development and metabolism (36, 37). Our results indicated that the destruction of cell structure and abnormal activity in sepsis may be consistent with the fact that the destruction of the actin skeleton is caused by abnormal disulfide bond formation in disulfidptosis, which still needs to be explored further.

In our evaluation of the connection between ACSL4 co-expression genes and sepsis, we identified GK, CLEC4E, ACSL1, TGFBR3, ADAM9, AZI2, BCAT1, and CYP1B1 as overlapping genes. Among these genes, CLEC4E (also called as Mincle) is a C-type lectin receptor, which can ameliorate bacterial pneumonia by regulating neutrophil phagocytosis and extracellular trap formation (38). There is a lack of definitive research data on the specific role of CLEC4E in disulfidptosis or sepsis. However, considering it as a C-type lectin family members may participate in immunoregulation, it may be associated with sepsis in the inflammatory response. It has been demonstrated that increased lipopolysaccharide can activate macrophage Clec4e, which, in turn, promotes macrophage proliferation and inflammatory response activation (39). Studies have shown that in lung tissue, M1 and M2 macrophages as well as neutrophils of mice in the pulmonary group exhibited high expression of Clec4e. The expression of Clec4e in neutrophils and macrophages may serve as a marker for the various components that contribute to ARDS (40). Conclusions from the literature and publicly available transcriptome data suggest that ACSL1 is probably involved in neutrophil inflammasome activation during sepsis (41). ACSL1, similar to ACSL4, is also involved in fatty acid metabolism, but its specific association with disulfidptosis remains unclear. Based on literature as well as inferences from co-expression analyses in other literature, ACSL1 and ACSL4 expression levels were significantly higher in fatal sepsis cases (41). Previous studies have shown that ACSL1 plays a proinflammatory role in monocytes/macrophages and neutrophils in sepsis (42). Transforming growth factor-β type III receptor (TGFBR3) is involved in transforming the TGF-β signaling pathway, which plays an important role in cell growth, differentiation, and apoptosis. Abnormal activation or inhibition of TGF-β may affect the survival state of cells and indirectly correlate with disulfidptosis. However, there is a lack of related research. Another bioinformatics analysis found that TGFBR3 expression was downregulated in septic blood samples (43). TGFBR3 is lowly expressed in sepsis and lncRNA H19 could decrease LPS-induced proinflammatory cytokine production to ameliorate ALI by targeting TGFBR3 (44). Cytochrome P450 1B1 (CYP1B1), a member of CYP superfamily, involved in drug metabolism and detoxification. Although its direct relationship to disulfidptosis is unclear, abnormal function of CYP1B1 may affect the response and detoxification ability of cells to inflammation, thereby indirectly affecting cell survival status. CYP1B1 was high expression in Wnt5A-associated inflammatory, cardiovascular diseases, and sepsis (45). BCAT1 is involved in the metabolism of branched-chain amino acids, and its dysfunction may be related to the imbalance of amino acid homeostasis. Although its direct relationship to disulfidptosis is unclear, an imbalance in amino acid homeostasis may affect the survival state of the cell. The potential role of BCAT1 in sepsis is also suggested by studies showing that elevated blood BCAA improves the catabolic effect of LPS on skeletal muscle protein synthesis in a mouse model of sepsis, which may provide a survival advantage in response to bacterial infection (46). However, the other ACSL4-related co-expressed genes have not been reported so far, and further studies are still needed to clarify their related mechanisms. Four of the eight overlapping genes are closely related to sepsis. In addition, the TF-miRNA coregulatory interactions composed of these genes were associated with sepsis. Transcription factor SP1 ameliorates sepsis-induced myocardial injury and intestinal barrier dysfunction (47, 48). Moreover, silencing miR-543 could inhibit LPS-induced inflammation and apoptosis to alleviate sepsis-induced AKI via targeting Bcl-2 (49). All those research studies were prompted that ACSL4 may affect sepsis through the interconnection between genes.

Similarly, we also analyzed the relationship between MYL6 co-expression genes and sepsis, a total of 10 overlapping genes were obtained, including ESYT1, ANXA1, CKLF, CD63, GMFG, HAT1, LDHA, SLPI, COX7B, and S100A8. Many studies have shown that most of these genes were related to sepsis. For instance, Annexin A1 (ANXA1) is one of the calcium-dependent phospholipid-binding protein family, which plays a role in anti-inflammatory and pro-apoptotic effects and can reduce tissue damage by downregulating early inflammation. Circulating AnxA1 levels were increased in a subgroup of patients with sepsis (50). Other studies have suggested Annexin A1 could afford protection effect against LPS-induced AKI by inhibited phosphorylation of PI3K and AKT and downregulated the expression of NF-κB (51). CD63 is a member of the tetraspanin protein family that is widely expressed on exosomes and as exosome markers, which is associated with extracellular vesicles and apoptosis, may be involved in the process of cell death. Recent studies have found that elevated exosomal CD63 levels are associated with the severity of organ failure and predict the mortality of severe sepsis patients (52). Lactate dehydrogenase A (LDHA) downregulation can improve immune function in sepsis by inhibiting glycolysis in polymorphonuclear neutrophils contributing to neutrophil immunosuppression (53). Secretory leukocyte protease inhibitor (SLPI) regulates inflammation response by downregulating the NF-κB pathway, which may modulate immunity response by inhibiting lymphocyte proliferation and the formation of NETs. Moreover, the expression of human plasma SLPI in sepsis was increased, which was related to the degree of organ dysfunction (54). Cyclooxygenase 7B (COX7B), one component of complex IV of the mitochondrial electron transport chain, regulating COX7B can significantly enhance mitochondrial performance of immune cells in sepsis (55). S100A8/A9, belonging to a calcium-binding protein, was massive secreted by activated neutrophils during infection and inflammation, and inhibiting S100A8/A9 could improve sepsis-induced myocardial dysfunction by reducing systemic inflammation and restoring myocardium mitochondrial function (56). Unfortunately, there is a lack of studies correlating disulfidptosis with these genes. We hypothesized that the co-expression genes in our study may be indirectly affected by disulfidptosis in the following ways: (1) Metabolic regulation: For example, LDHA affects cellular energy metabolism by participating in the glycolysis process and then affects the occurrence of disulfidptosis; (2) Regulation of apoptosis: For example, ANXA1 has a pro-apoptotic effect and may affect disulfidptosis by regulating the apoptotic pathway; (3) Cytoskeleton and morphological stability: as GMFG affects cytoskeletal structure and intercellular junctions, it may affect disulfidptosis by affecting cell morphology and stability. MYL6 co-expressed genes (ESYT1, ANXA1, CKLF, CD63, GMFG, HAT1, LDHA, SLPI, COX7B, and S100A8) may be associated with disulfidptosis and sepsis through different mechanisms. They may regulate the cell metabolism, cell apoptosis, and inflammation process to influence the progress of the disulfidptosis and sepsis. However, further studies are needed to elucidate the specific molecular mechanisms and interactions. Moreover, in the TF-miRNA coregulatory interactions composed of these genes, the top three hsa-miR-23a-3p was considered to regulate NF-κB signaling pathway-related HDAC7/ACTN4 to influence the progression of neurovascular-related sepsis-induced cardiomyopathy (57). Previous studies suggested that ACSL4 and MYL6 co-expression genes were closely associated with inflammatory response, immunosuppression, cell function metabolism, and other aspects in the progression of sepsis, which was consistent with our conjecture and research.

Finally, we designed a validation study to investigate the differential expression of MYL6 and ACSL4 in sepsis-induced acute lung injury. The mRNA levels of MYL6 and ACSL4 in the LPS group were increased *in vitro* and *in vivo* experiments of sepsis-induced ALI models. Previous studies have identified MYL6 as a disulfidptosis-related gene through bioinformatics, which may be involved in the occurrence and development of diseases such as Alzheimer’s and non-alcoholic fatty liver (58, 59). However, it is not yet clear how it regulates the mechanism of disulfidptosis. This article identifies for the first time MYL6 as a disulfidptosis-related gene involved in sepsis-induced acute lung injury. In addition, this article reports for the first time on ACSL4 as a disulfidptosis-related gene involved in sepsis-induced acute lung injury. At the same time, some indexes of disulfidptosis showed different trends in the LPS group, but the NADP+/NADPH ratio increased. We speculate that this may be because the composition of lung tissue is more complex and has different regulatory mechanisms from RAW264.7. The findings suggested that disulfidptosis might be a major factor in lung injury brought on by sepsis. This part of the scientific problem is also the direction of our next research. Additionally, how ACSL4 and MYL6 regulate the involvement of disulfidptosis in the occurrence of acute lung injury in sepsis is also our next research direction.

With MR analysis, we also carried further and discovered a causal relationship between MYL6 and sepsis (a 28-day death in critical care). In this study, the SNP (rs35436573) in the MYL6 gene was found to be associated with the survival rate of sepsis by MR-related analysis, suggesting that genetic variation in the gene may influence individual responses to sepsis. The specific mechanism is unclear; however, it may involve genes in regulating immune responses, the role of inflammation control, or other related physiological processes. In addition, a series of bioinformatics analyses and related experimental studies in this study suggested that MYL6 was associated with ALI in sepsis. The expression of the MYL6 gene in lung tissue affects the regulation of inflammatory response or the ability of lung tissue to cope with infection. ALI is a serious complication of sepsis. As SNPs affect gene expression and function, we can speculate that SNPs in the MYL6 gene may affect the progression of sepsis and the occurrence of ALI by affecting the expression of these genes in sepsis. If changes in the expression of the MYL6 gene result in a more severe inflammatory response or lung injury, then they may reduce survival in sepsis. The SNP rs35436573 is located on chromosome 12 of the human genome (specifically at Chromosome 12:56159225) and is within the intronic region of the MYL6 gene. Previous studies have not investigated the function of this SNP. In this study, we used online tools such as VannoPortal, HaploReg, and RegulomeDB to comprehensively predict the relevant functions of this SNP (10, 11, 60). The RegulomeDB probability score ranges from 0 to 1, with 1 representing the most likely regulatory variant. The RegulomeDB score is based on continuous values derived from functional genomics features and experimental sources, such as ChIP-seq signals, Dnase-seq signals, and DeepSEA scores (61). By using RegulomeDB, we found that rs35436573 has a RegulomeDB grade of 1a, with a score of 0.836, suggesting that rs35436573 plays an important role in the MYL6 gene. Shown by RegulomeDB online, in lung tissue and the A549 cell line, the ChIP-seq analysis shows there is a peak with the transcription factor POLR2, indicating that the transcription factor POLR2 may be binding and playing a role near this SNP. Additionally, in the A549 cell line and lung tissue, this SNP may be associated with active transcription start sites (TSS) and upstream of the flanking TSS. Furthermore, this SNP may function by altering the binding affinity of transcriptional regulators ESR1 and ESR2 by changing their motifs, thereby regulating transcription factor binding. Moreover, expression quantitative trait locus (eQTL) studies suggest that this SNP may affect the expression of RPS26 or IKZF4 in lung tissue. In summary, rs35436573 may affect the expression level of target genes by affecting transcription factors.

Based on the analysis using VannoPortal and HaploReg online tools, we also discovered that in lung tissue, the rs35436573 mutation can modulate histone modifications such as H3K27ac, H3K4me2, H3K4me3, H3K79me2, H3K9ac, and H4K20me1. This affects the affinity between histones and the DNA double helix, thereby altering the nucleosome structure as well as the loose or condensed state of chromatin. It can also regulate gene expression by influencing the affinity of other transcription factors to structural gene promoters. In particular, H3K4me3 serves as a promoter marker and is associated with gene activation. It is primarily enriched in promoter regions near TSS. Similarly, H3K27ac is also related to gene activation and is mainly enriched in enhancer and promoter regions. When H3K27ac modifications are simultaneously enriched in enhancer regions, these enhancers become activated, promoting gene expression. Furthermore, based on the analysis of the changes in allele-specific binding affinities of motifs for important transcription regulators, it is indicated that the rs35436573 mutation may alter 14 transcription factor motifs, modulating the binding strength at transcription factor sites to exert its effects. The transcription factors affected include BHLHE40, DIDO1, PAX1, ZNF143, NR2C1, KLF1, EGR1, IRF5, BRCA1, TCF12, SRF, and KLF2. Additionally, ChIP-seq analysis of transcription factor binding peak overlaps suggests that this SNP may exert biological effects by influencing the binding of transcription regulators, such as POLR2A, EP300, ESR1, and SPI1. However, current evaluations using SIFT, PolyPhen-2, and other online tools have not identified any direct impact on protein stability. Further studies are needed to investigate the biological role of this SNP. Therefore, based on functional predictions of the SNP, this study speculated that SNP (rs35436573) variation in MYL6 may affect the expression level of MYL6, resulting in a more severe inflammatory response or lung injury, then they may promote the progression of sepsis and the occurrence of acute lung injury. Consistent with earlier results from our study, it is clear that MYL6 overexpression plays a major role in the pathophysiology of sepsis. Therefore, targeted regulation of those genes may be a new target for sepsis therapy.

Nowadays, bioinformatics analysis is widely used to forecast new biomarkers and offer new perspectives on the pathophysiology of disease as it has demonstrated outstanding performance in clinical diagnosis. In the present study, we used ROC curve analysis to evaluate the potential diagnostic value of ACSL4 and MYL6 in sepsis. Both ACSL4 and MYL6 exhibited high AUC values in training datasets. Similar results are also shown in validation datasets. The credibility of results was confirmed by AUC values consistently exceeding 0.75, indicating the dependability of the diagnostic prediction and the potential for its effective adoption into clinical practice. The above results suggested that ACSL4 and MYL6 may be the new biomarkers to predict and early diagnose sepsis. Nevertheless, our research still has some limitations. More rigorous and scientific basic experiments have not been conducted to investigate and validate the possible molecular mechanism of DRGs in sepsis. In addition, more samples are required to assess the precision of the key genes as novel biomarkers. In the following research, we will address these constraints.

## 5 Conclusion

This study established an association between sepsis-induced ALI and disulfidptosis. The key genes, including ACSL6 and MYL6, were identified, and their related functions and genetic regulatory networks were constructed. Moreover, the mRNA levels and AUC values of key genes were verified. Some results of bioinformatics analysis were verified by basic experiments. Finally, we explored the causal relationship between MYL6 and sepsis. These results involved the identification and validation of disulfidptosis-related genes in sepsis-induced ALI, which may be helpful for the diagnosis and treatment of septic lung injury.

## Data Availability

The original contributions presented in the study are included in the article/Supplementary material, further inquiries can be directed to the corresponding authors.

## Footnotes

^1^https://www.ncbi.nlm.nih.gov/geo/

^2^https://www.ncbi.nlm.nih.gov/geo/geo2r/

^3^https://cibersortx.stanford.edu/

^4^https://genemania.org

^5^https://metascape.org/gp/index.html#/main/step1

^6^http://www.ehbio.com/test/venn/#/

^7^https://www.networkanalyst.ca/

^8^http://shiny.cnsgenomics.com/mRnd/

^9^https://regulomedb.org/

^10^https://pubs.broadinstitute.org/mammals/haploreg/haploreg.php

^11^http://mulinlab.tmu.edu.cn/gwas4d

^12^https://sift.bii.a-star.edu.sg/

^13^http://genetics.bwh.harvard.edu/pph2/

^14^https://www.r-project.org/
