# Supplementary material for: Identification and verification of disulfidptosis-related genes in sepsis-induced acute lung injury
Source: Front Med (Lausanne). 2024 Aug 28;11:1430252. doi: 10.3389/fmed.2024.1430252 (PMC11389619; doi:10.3389/fmed.2024.1430252)
Supplement: Supplementary file 1 [file Data_Sheet_1.pdf]

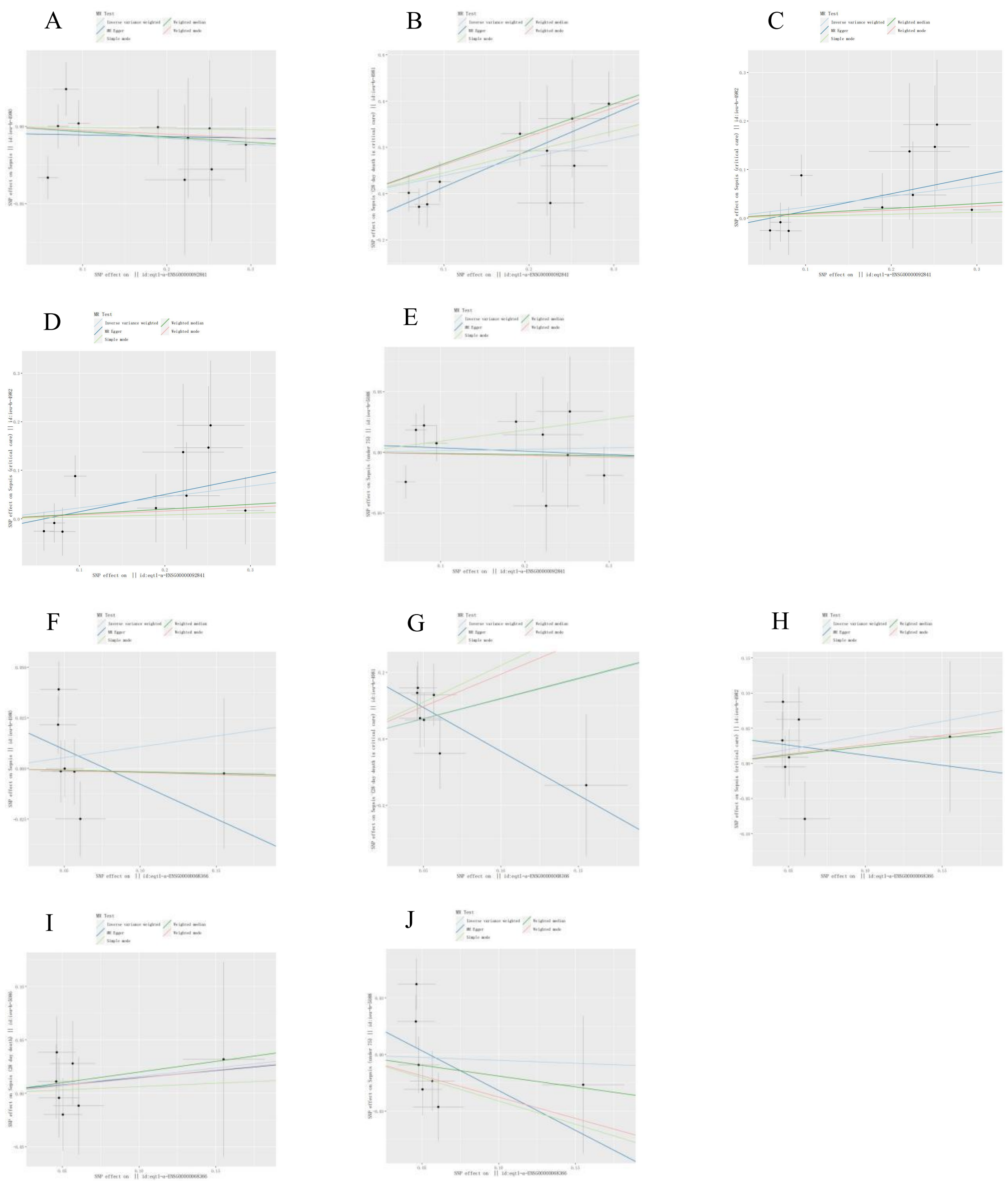

Figure S1 Scatter plots of the risk of genetic association between sepsis and MYL5/ACSL4 by MR (The slopes of each line represent the causal association for each method). (A-E: MYL6 on ieu-b-4980, ieu-b-4981, ieu-b-4982, ieu-b-5086, ieu-b-5088, respectively; F-J: ACSL4 on ieu-b-4980, ieu-b-4981, ieu-b-4982, ieu-b-5086, ieu-b-5088, respectively)

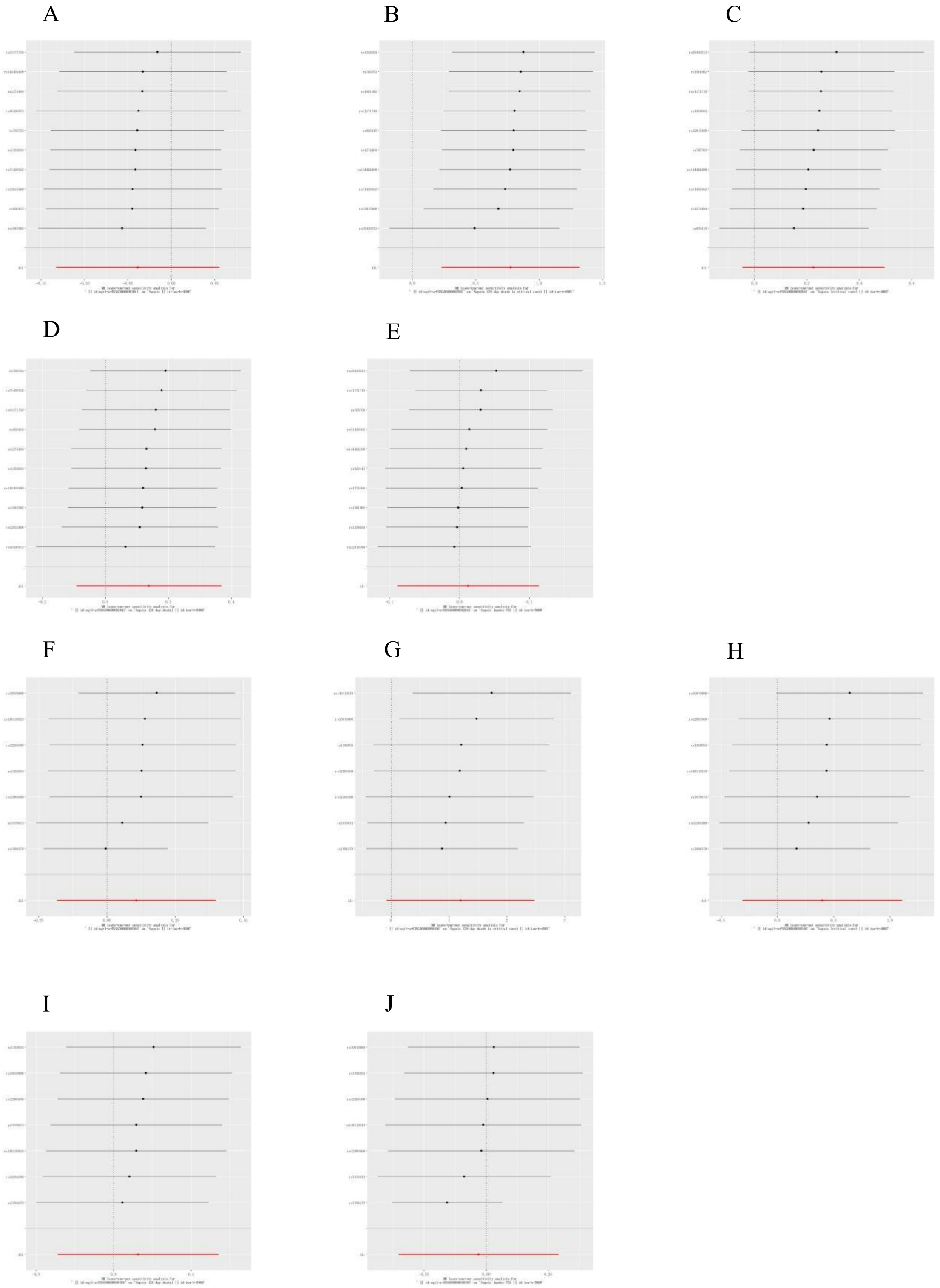

Figure S2 The result of 'Leave-one-out' method. (A-E: MYL6 on ieu-b-4980, ieu-b-4981, ieu-b-4982, ieu-b-5086, ieu-b-5088, respectively; F-J: ACSL4 on ieu-b-4980, ieu-b-4981, ieu-b-4982, ieu-b-5086, ieu-b-5088, respectively)

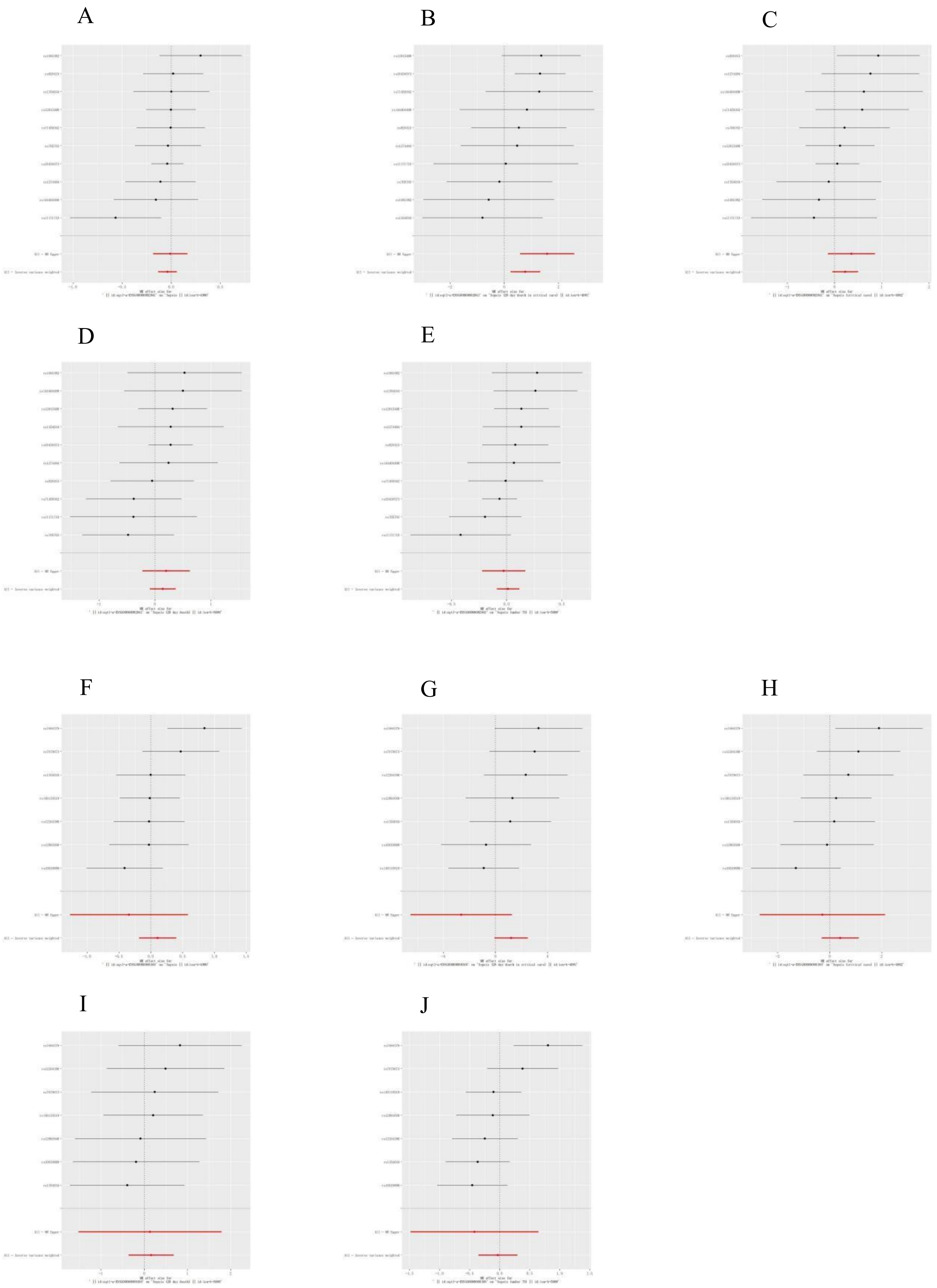

Figure S3 Forest plots of illustrating how exposures effect the risk of outcomes. Using each SNP separately, the causal relationship between exposures and outcomes is calculated in MR. (A-E: MYL6 on ieu-b-4980, ieu-b-4981, ieu-b-4982, ieu-b-5086, ieu-b-5088, respectively; F-J: ACSL4 on ieu-b-4980, ieu-b-4981, ieu-b-4982, ieu-b-5086, ieu-b-5088, respectively)

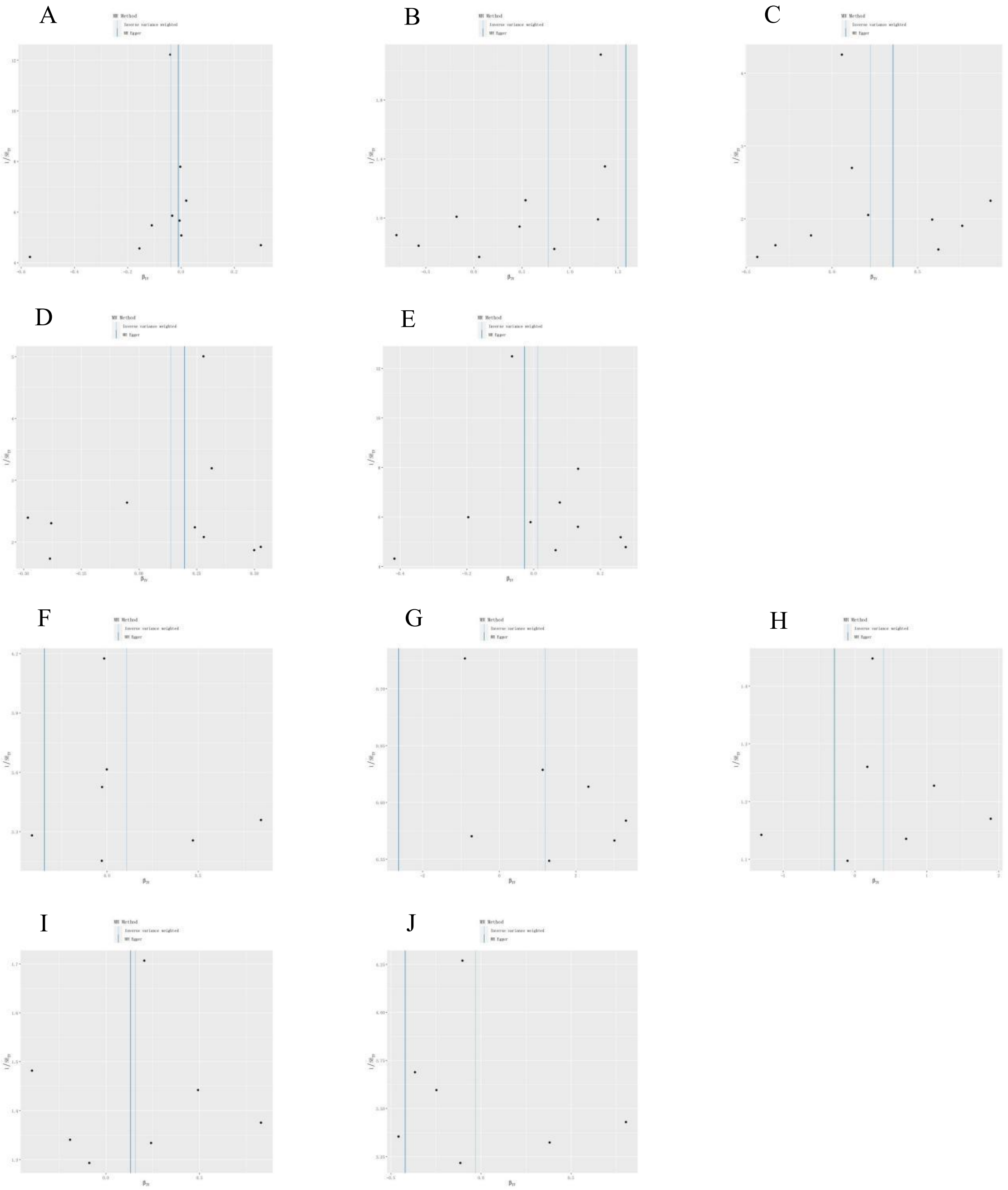

Figure S4 Funnel plots of individual variation effects for the instrument variables shown against the inverse of their standard error (A-E: MYL6 on ieu-b-4980, ieu-b-4981, ieu-b-4982, ieu-b-5086, ieu-b-5088, respectively; F-J: ACSL4 on ieu-b-4980, ieu-b-4981, ieu-b-4982, ieu-b-5086, ieu-b-5088, respectively)

Supplementary Table S1

Disulfidptosis-related genes(DRGs)			
ACTB	LRPPRC	PDLIM1	PRDX1
ACTN4	MYH10	RPN1	ACSL4
CAPZB	MYH9	SLC3A2	BAK1
CD2AP	MYL6	SLC7A11	TLN2
DSTN	NADPH	TLN1	FLNC
FLNA	NCKAP1	BRK1	MYL6B
FLNB	NDUFA11	RAC1	DBN1
GYS1	NDUFS1	CYFIP1	ACTN1
INF2	NUBPL	WASF2	MYH11
IQGAP1	OXSM	ABI2	
